# Generation of proliferating human adult hepatocytes using optimized 3D culture conditions

**DOI:** 10.1038/s41598-020-80019-4

**Published:** 2021-01-12

**Authors:** Sophie Rose, Frédéric Ezan, Marie Cuvellier, Arnaud Bruyère, Vincent Legagneux, Sophie Langouët, Georges Baffet

**Affiliations:** Univ Rennes, Inserm, EHESP, Irset (Institut de Recherche en Santé, environnement et travail)-UMR_S 1085, 35043 Rennes Cedex, France

**Keywords:** Cell growth, Cell growth, Transcriptomics, Hepatocytes, Cell signalling

## Abstract

Generating the proliferation of differentiated normal adult human hepatocytes is a major challenge and an expected central step in understanding the microenvironmental conditions that regulate the phenotype of human hepatocytes in vitro. In this work, we described optimized 3D culture conditions of primary human hepatocytes (PHH) to trigger two waves of proliferation and we identified matrix stiffness and cell–cell interactions as the main actors necessary for this proliferation. We demonstrated that DNA replication and overexpression of cell cycle markers are modulate by the matrix stiffness while PHH cultured in 3D without prior cellular interactions did not proliferate. Besides, we showed that PHH carry out an additional cell cycle after transient inhibition of MAPK MER1/2-ERK1/2 signaling pathway. Collagen cultured hepatocytes are organized as characteristic hollow spheroids able to maintain survival, cell polarity and hepatic differentiation for long-term culture periods of at least 28 days. Remarkably, we demonstrated by transcriptomic analysis and functional experiments that proliferating cells are mature hepatocytes with high detoxication capacities. In conclusion, the advanced 3D model described here, named Hepoid, is particularly relevant for obtaining normal human proliferating hepatocytes. By allowing concomitant proliferation and differentiation, it constitutes a promising tool for many pharmacological and biotechnological applications.

## Introduction

Hepatocytes are highly differentiated cells that carry out most of hepatic functions. In vivo, hepatocytes are normally long-lived quiescent cells but, contrary to most terminally differentiated cells, they retain their capability to proliferate, thus allowing liver regeneration in case of acute injury or loss of functional mass^1^. In vitro*,* the proliferation of adult primary human hepatocytes (PHH) has never been obtained and is still a major challenge.

To date, the proliferation of cultured primary hepatocytes has only been described with rodent hepatocytes^2–4^. PHH show limited survival in 2D primary culture and die 8–10 days after seeding. Moreover, they rapidly lose most of their differentiated functions, thus restricting metabolic, pharmacological and toxicological studies. Some relevant co-culture experiments with non-parenchymal liver cells^5,6^ or hepatocellular carcinoma cell lines^7,8^ as well as the addition of DMSO^9^ allow long-term survival and differentiation of primary rodent hepatocytes. Similarly, these conditions greatly improve the survival and functionality of human hepatocytes as well as the maintenance of some liver-specific functions^10–13^ but, in any case, do not enable proliferation. Recently, great interest has been granted to immortalization techniques by transfection with oncogenes to obtain proliferating hepatocytes. Such hepatocytes exhibited rapid expansion but rapidly de-differentiated and lost their liver functions as cultures progressed with uncontrolled proliferation^14–17^. Proliferating human hepatocytes have also been generated by reprogramming fibroblasts^18,19^ whereas some studies relied on the in vivo plasticity of adult hepatocytes to transdifferentiate into proliferative bipotent progenitor cells in response to chronic injuries to establish long-term organoid culture^19–23^.

Increasing numbers of 3D hepatocytes culture models have been described and extensively reviewed these last years^24,25^. These alternative systems have proven to be the most appealing strategy to maintain liver phenotype and thus long-term hepatic functionality and retaining the molecular signatures of PHH^26,27^. As it has been reported that 3D scaffolding geometry can modulate the functionality of hepatocytes and gene expression^28^, placing human papilloma virus (HPV) transfected human hepatocytes into a scaffold-based model increases phase I and II xenobiotic metabolism enzymes activities as well as expression of liver-specific proteins^14,29^. Interestingly, we and others have shown that increasing the stiffness of the matrix promotes enhancement of the phenotypic and functional maturation of the encapsulated hepatoma cells^30,31^. Proliferation of human hepatic progenitor cells from healthy donors or pediatric patients and redifferentiation into metabolically competent cells are boosted by three-dimensional culture^32^. Several interesting studies also report work carried out on PHH cultured in 3D. An increase of metabolic capabilities was observed when cultured in hybrid hydrogels^33,34^ while microfluidic systems improved both viability and hepatic-specific functions during up to 14 days of culture^35^. Liver bioreactor systems also retained high viability and biotransformation capabilities of PHH allowing the detection of metabolites from slowly metabolized drugs at chronic doses for up to 2 weeks^36–38^. Normal human hepatocytes seeded into ultra-low attachment plates remained phenotypically stable and retained morphology, viability and hepato-specific functions for culture periods of at least 5 weeks without any scaffold^26,27,39^. Critically, none of the cited culture systems using non-transfected cells enabled the proliferation of normal human hepatocytes in vitro, which is essential for generating a source of normal liver parenchymal cells.

We describe for the first time a well-defined model of PHH collagen culture, named Hepoid, defined by PHH organized as characteristic hollow spheroids and able to perform two waves of proliferation while maintaining long-term survival, hepatic differentiation, xenobiotic biotransformation and cell polarity characteristic of normal human hepatocytes. Furthermore, our results show that PHH can re-initiate a new cell cycle after transient MAPK MER1/2-ERK1/2 signaling pathway inhibition. The advanced 3D model described here is particularly relevant for obtaining non-transformed proliferating human hepatocytes in primary culture. It will provide a long awaited and promising starting point for further biotechnological developments.

## Results

### Optimized 3D collagen culture conditions enable spheroid formation, polarization and long-term survival of adult human hepatocytes

**Figure 1:**
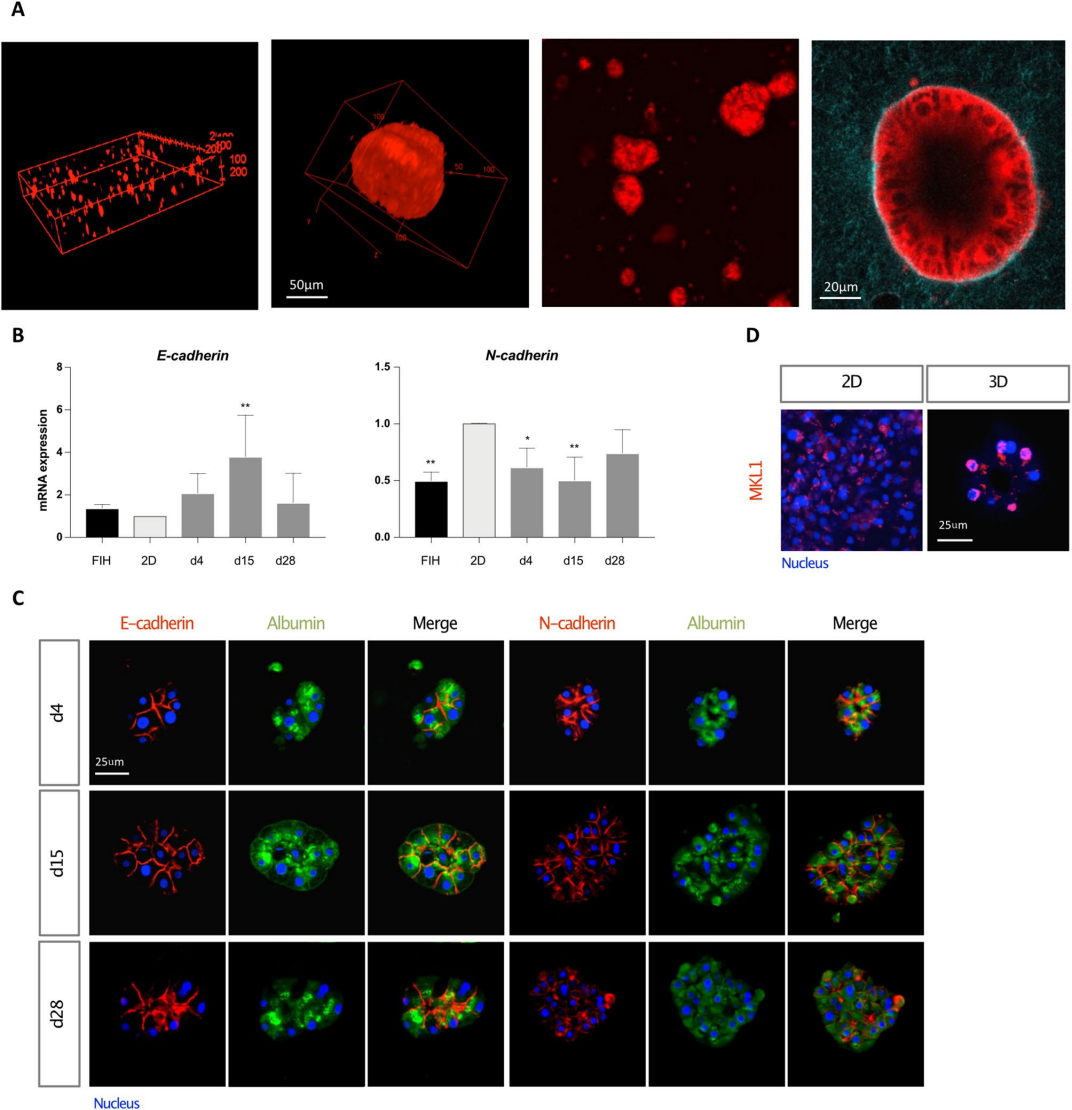
Optimized 3D collagen culture conditions promote spheroid formation and the maintenance of hepatic functions over time. (**A**) TPEF images of Hepoid after 8 days of culture. 3D reconstruction (xyz) of (left) 250 μm and (middle left) 100 μm depth images stack (TPEF stack) at 10× and 60× magnification, respectively; (middle right) and (right) are TPEF images taken at 20× and 60× magnification, respectively. (**B**) Quantification of the gene expression patterns by RT-qPCR of *E-cadheri*n and *N-cadherin* in FIH (black), 2D cultures (light grey) and 3D cultured PHH (dark grey), 4 (d4), 15 (d15) and 28 days (d28) after seeding. The results are from at least three independant experiments and are expressed according to the 2D culture level (*p < 0.05, **p < 0.01, ***p < 0.001, ****p < 0.0001, one-way ANOVA test, GraphPad Prism v.7.0). (**C**) Immunostaining of E-cadherin or N-cadherin (red), Albumin (green) and nuclei (blue) in PHH spheroids at d4, d15 and d28. (**D**) Immunostaining of MKL1 (pink) and nuclei (blue) in 2D and 3D cultures. Scale bar = 25 μm.

Our previously published work has shown that the rigidity of a collagen I matrix influenced the cell phenotype and induced the proliferation and spheroid clustering of the human hepatocarcinoma cells Huh7^30^. By following the same experimental approach, PHH failed to come together and the cells stayed isolated due to their lack of motility (see Supplementary Fig. S1A online). In order to promote the establishment of cell–cell interactions between PHH, we seeded the cells into low attachment plate (LAP) before their inclusion into the collagen matrix. The clumps remained grouped and formed small clusters in the collagen matrix (see Supplementary Fig. S1B online). The cultures were stimulated by growth factors (EGF, HGF, ITS) known to play a role in rodent hepatocyte proliferation in vitro^3^. To examine the organization of cells in 3D collagen gels, we performed TPEF (Two-Photon Excitation Fluorescence) imaging and Z stack reconstruction at day 8, showing a homogeneous distribution of large cell aggregates (Fig. 1A). At high magnification (60×), the cluster of PHH appears as a hepatosphere bordered by a single layer of well-organized cells forming an acini-like structure with a hollow lumen, characteristic of the organization of our spheroids (Fig. 1A). Analyzed by qRT-PCR, 15 days cultivation time of PHH in 3D collagen gels was associated with increased expression of the epithelial marker *E-cadherin* while expression of the mesenchymal marker *N-cadherin* remained stable throughout the culture (Fig. 1B). It is important to emphasize that two stem cell markers, *Cytokeratin 9* and *EpCAM*, could not be detected at the mRNA and protein levels in 3D cultures even at the longest time thus demonstrating the mature phenotype of the cells (results not shown).

Moreover, immuno-detection in spheroids showed distinct N- and E-cadherin locations at the apico, lateral- and basal-membranes in agreement with their respective specific localization in the human liver demonstrating the epithelial characteristics of the spheroids (Fig. 1C). The localization of these two proteins have been reported with a zonal distribution: E-cadherin being described as more expressed in periportal hepatocytes while N-cadherin is more enriched in perivenous mouse hepatocytes^40^. Interestingly, the transcription factor MKL1/MRTF-A showed a specific nuclear localization only in 3D cultures (Fig. 1D), in accordance with its recognized force-mediated dependent activity^41^. The absence of apoptosis in Hepoid was analyzed by cleaved caspase-3 immunolocalization which was not detected at basal level but only after a 24 h cisplatin treatment (see Supplementary Fig. S1C online).

### First evidence of adult human hepatocytes proliferation induced by 3D microenvironment: highlighting of two waves of proliferation

**Figure 2:**
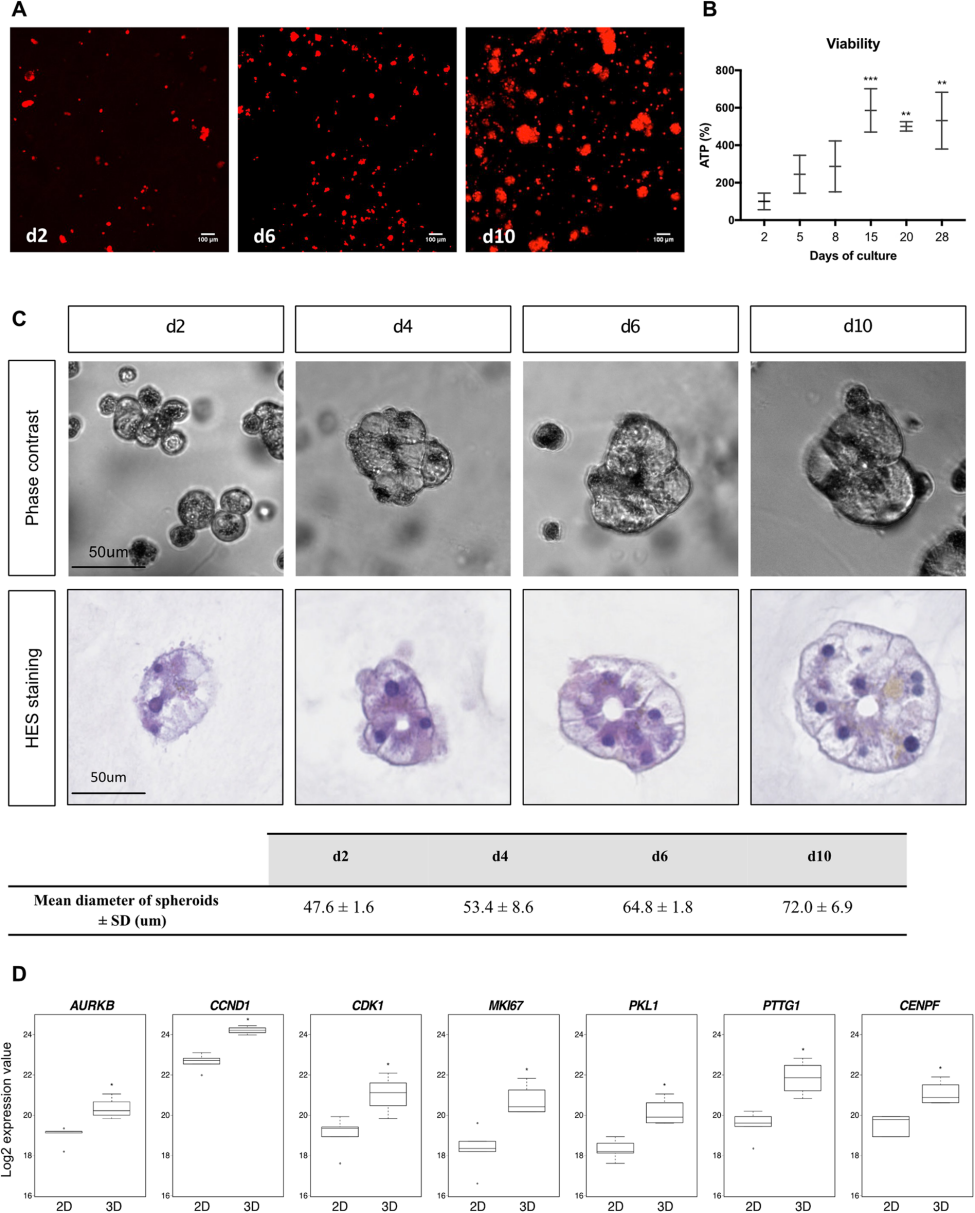
The size of spheroids sharply increases and correlates with the expression of proliferation genes. (**A**) TPEF images of 3D cultured PHH at d2, d6 and d10 of culture (10× magnification, z projection). Scale bar = 100 μm. (**B**) The viability was assessed by measuring the cellular ATP content in 3D cultured PHH at different times of culture (n = 3) (Mean ± SD, **p < 0.01, ***p < 0.001, one-way ANOVA test). (**C**) Phase-contrast imaging and HES staining of spheroids at d2, d4, d6 and d10 of culture. Scale bar = 50 μm. Quantification of the mean diameter size of spheroids during the time of culture. Diameters of spheroids are from three independents experiments (Mean ± SD). (**D**) Boxplots extracted from transcriptomic analysis of the expression of the main genes involved in the proliferation of epithelial cells (*AURKB, CCND1, CDK1, MIK67, PKL1, PTTG1, CENPF*). (*p < 0.05, Mann–Whitney U test).

**Figure 3:**
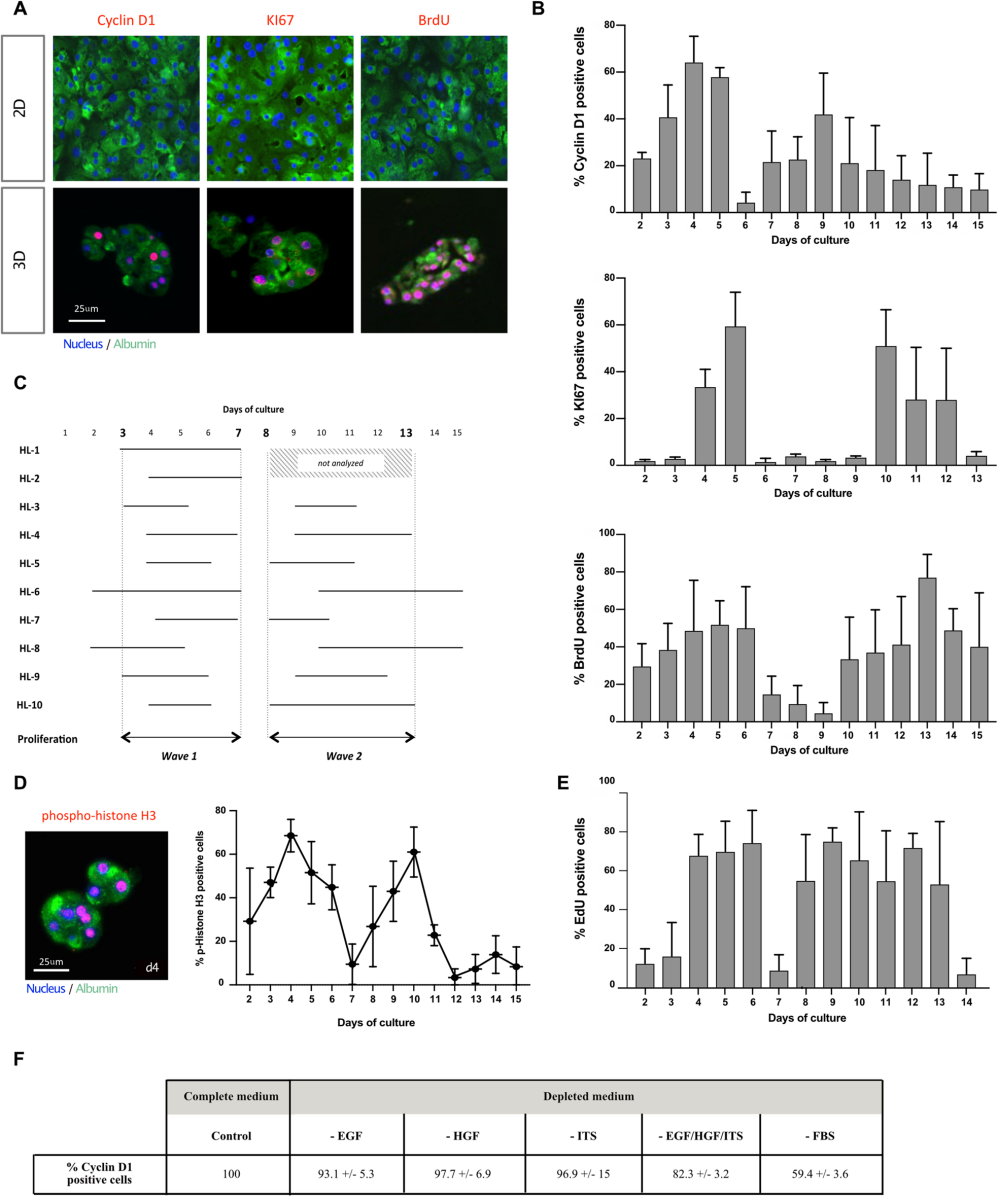
3D cultured PHH perform spontaneously two waves of proliferation. (**A**) Illustration of the staining of two markers of proliferation, Cyclin D1 and KI67, and the incorporation of BrdU (red) at d4 in the PHH cultured in 2D or in 3D. Albumin (green) was always stained in parallel to attest the hepatic phenotype. Nuclei (Dapi) were in blue. Scale bar = 25 μm. (**B**) Two waves of proliferation were observed in Hepoid during the first 2 weeks of culture as shown by the quantification of positive Cyclin D1 and KI67 cells and of the incorporation of BrdU. (**C**) These two waves of proliferation were observed in all cases of PHH analyzed (HL-3 to HL-10). The common proliferation waves always occurred between days 3 and 7 for the first wave and between days 8 and 13 for the second. For cases HL-1 and HL-2, quantification for the KI67 labeling index was done only during the 1st week. (**D**) (Left) Fluorescent immunostaining of phospho-histone H3 (red), a marker for mitotic cells, Albumin (green) and nuclei (blue) in Hepoid at d4 after 24 h of treatment with colcemid (1 μm). Scale bar = 25 μm. (Right) Mitotic index quantified by phospho-histone H3 positive cells in spheroids at the indicated time in the figure, after treatment with colcemid. (**E**) The incorporation of EdU was used to quantify the rate of proliferation of cryopreserved PHH cultured in 3D. (**F**) Percentage of proliferating PHH (Cyclin D1/Albumin positive cells) seeded in collagen gels at 1.5 mg/ml with complete culture medium (Control) or in medium depleted in EGF, HGF, ITS or FBS.

We examined the formation and evolution of PHH spheroids during the first 2 weeks of culture. Two days after 3D seeding in the collagen gels, PHH appeared as small clusters of heterogeneous sizes, consisting of round cells without membrane extension or particular organization (Fig. 2A,C). However, at days 6 and 10, the PHH spheroids were more numerous and larger, indicating that 3D cultures in collagen matrix should influence the formation and extension of clusters during culture. Quantification studies showed a 4.5-fold increase in TPEF-autofluorescent cell signal between days 2 and 10 (Fig. 2A) and ATP levels in spheroids were significantly increased during the first 15 days and remained at high level thereafter (Fig. 2B). At day 6, quantification of the number of nucleus in Hepoid showed a 1.9 ± 0.2-fold increase compared to the culture of isolated PHH (quantification from Supplementary Fig. S1A,B online). Using phase-contrast imaging or after HES (hematoxylin, eosin, saffron) staining, the first striking observation was that all the spheroids were formed from only one layer of cells regardless of the time of culture, forming a hollow form of spheroid with a characteristic lumen inside that we defined as Hepoid. The average diameter of the spheroids was gradually increased as a function of time of culture (Fig. 2A,C) and, as we could observe by time-lapses, the spheroids increased in size from day 3 to day 6 and the isolated PHH did not migrate in the collagen gel (see Supplementary Fig. S2 online). The proportion of spheroids with a diameter less than 60 μm decreased with time while those with a diameter greater than 60 μm gradually increased (see Supplementary Fig. S3 online). Size quantification indicated an increase from 47.62 μm ± 1.61 μm at day 2, to 72.04 μm ± 6.92 μm at day 10 corresponding approximately to a threefold increase of the spheroid volume (Fig. 2C). Next, by transcriptomic analysis performed as described in material and methods, we analyzed the expression of specific cell cycle markers^42^ (*AURKB*, *CCND1*, *CDK1*, *MKI67*, *PKL1*, *PTTG1* and *CENPF*) and we showed that the mRNA expressions of all these genes were significantly increased in 3D cultured PHH after 15 days than in 2D cultures (Fig. 2D). The proliferation was further confirmed by the quantification of Cyclin D1, KI67 and BrdU positive cells, markers of late G1 and S phases during the two first weeks of culture of PHH isolated from 10 different donors (Table 1, Fig. 3).

**Table 1:** Clinical characteristics of human liver (HL) samples.

Case	Age	Gender	Liver pathology	Smoker	Diabetic
HL-1	68	F	Cholangiocarcinoma	Yes	No
HL-2	66	F	Liver mestatasis from colorectal carcinoma	No	No
HL- 3	72	F	Liver mestatasis from colorectal carcinoma	No	No
HL-4	56	M	Cholangiocarcinoma	No	No
HL-5	59	M	Liver mestatasis from colorectal carcinoma	No	No
HL-6	60	F	Liver mestatasis from colorectal carcinoma	No	No
HL-7	78	M	Liver mestatasis from stromal tumor	No	Yes
HL-8	60	M	Liver mestatasis from colorectal carcinoma	No	No
HL-9	43	F	Polycystic liver disease	No	No
HL-10	58	F	None	Yes	Unknown

Albumin was taken as a positive control to validate the hepatocyte phenotype of the enumerated cells. Our results demonstrated the proliferation of PHH in vitro, objectivized by the high proportion of Cyclin D1, KI67 and BrdU positive cells between days 2 and 15 (Fig. 3A,B). Hepatocytes undergo two waves of proliferation between days 2 and 7 and between days 8 and 15. More precisely, during the first week of culture, the average cumulative index of the S phase markers, KI67 and BrdU, reached 280% (± 60%) and 220% (± 40%), respectively. Only small differences in the kinetics of expression of proliferation markers could be observed, depending on the donor (Fig. 3C). The two successive waves of proliferation were also demonstrated by phospho-histone H3 immunostaining that quantify the proportion of cells in M phase (Fig. 3D). Thereafter, no positive nuclei were detected in the Hepoid (see Supplemental Fig. S4 online), as at no time in 3D cultures of isolated PHH (results not shown). Cyclin D1, KI67 or BrdU positive hepatocytes could not be detected in conventional 2D cultures although the PHH were stimulated with the same growth factor cocktail (results not shown). Interestingly, we have validated that cryopreserved PHH were also able to proliferate when cultured as Hepoid (Fig. 3E). To investigate the role of growth factors in the proliferation of 3D cultured PHH, we have depleted the culture medium of either EGF, HGF, ITS or FBS. The results showed that each depletion only partially decreased the proliferation by up to 41% in absence of FBS, showing that PHH could proliferate in 3D gels even in the absence of these additives (Fig. 3F). We also evaluated the effect of the collagen stiffness on PHH proliferation. While a lower collagen I concentration had no impact on hepatocyte proliferation, we found that increasing the concentration clearly inhibited hepatocyte proliferation. Indeed, the proliferation estimated by Cyclin D1 expression raised 105% ± 19.2% in 0.75 mg/ml collagen gels while it dropped to 50.2% ± 22.7% and 18.1% ± 1.5% at 3 mg/ml and 4 mg/ml concentrations, respectively (1.5 mg/ml collagen gels taken as control). As a consequence, at day 6, the number of nucleus was dramatically decreased when PHH were cultured in 3 and 4 mg/ml collagen gels (results not shown). Incubation of hepatocytes in LAP is a crucial step for establishing inter-cellular contacts and enabling the formation of aggregates, which will be included in the collagen matrix. Therefore, we showed that PHH embedded directly into collagen gels without prior establishment of cellular interactions (seeding without passage in LAP), PHH maintained in LAP and PHH cultured in conventional 2D cultures, had low or even zero proliferation rates (17.2% ± 7.1%, 9.2% ± 1.9% and 0%, respectively) compared to Hepoid. All these results demonstrated that cell–cell interactions, as well as the controlled stiffness, are the crucial factors inducing the proliferation of PHH.

### The MEK1/2-ERK1/2 signaling pathway plays a major role in the proliferation of Hepoid

**Figure 4:**
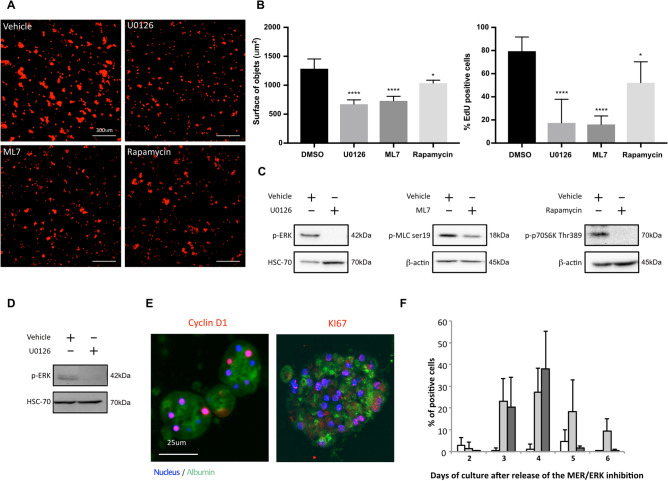
Implication of MEK1/2-ERK1/2 signaling pathway in the proliferation of Hepoid. Quantification of PHH proliferation after 6 days of culture in the presence of U0126 (20 μM), ML7 (20 μM), Rapamycin (10 nM) or vehicle (DMSO, 0.1%) (**A**–**C**). (**A**) TPEF images (left) and (right) quantification of the surface of Hepoid performed using ImageJ software. Scale bar = 300 μm. (**B**) Quantification of EdU incorporation during 24 h in the presence of inhibitors or vehicle. The results are expressed according to the DMSO-treated culture (*p < 0.05, **p < 0.01, ***p < 0.001, ****p < 0.0001, one-way ANOVA test). (**C**) Control of phospho-ERK, phospho-MLC on Serine 19 and phospho-P70 on Threonine 389 inhibition in the presence of U0126, ML7 and Rapamycin, respectively (+), or vehicle (−). Transient inhibition of the MEK1/2-ERK1/2 pathway was performed during 48 h by U0126 (20 μM) in 3D cultured PHH after the two waves of proliferation (**D**–**F**). (**D**) Phospho-ERK inhibition in the presence of U0126 (+) or vehicle control (−). Controls were done in presence of 0.1% DMSO. (**E**) Cyclin D1 or KI67 (red), Albumin (green) and nuclei (blue) staining. Scale bar = 25 μm. (**F**) Quantification of Cyclin D1 (light grey) and KI67 (dark grey) positive cells after release of the inhibition. Cyclin D1 was quantified in cells treated vehicle (white). No KI67 positive cell has been detected after treatment with the vehicle.

To evaluate the role of the MEK1/2/ERK1/2, PI3K and MLCK signaling pathways involved in regulating cell cycle progression of many cell types, including normal and transformed hepatocytes^2,30,43,44^, we conducted experiments in presence of specific inhibitors of these pathways: U0126 for MEK1/2/ERK1/2, Rapamycin for PI3K, and ML7 for MLCK. After 6 days of culture in the presence of inhibitors, we quantified the TPEF-autofluorescent cell signal and showed a 2-fold decrease after MEK1/2/ERK1/2 and MLCK inhibition while PI3K inhibition seemed to have a lesser impact on PHH proliferation (Fig. 4A). These results were confirmed by the quantification of EdU incorporation, which decreased significantly after the inhibition of MEK1/2/ERK1/2 and MLCK (Fig. 4B). As expected, U0126, Rapamycin and ML7 abolished the phosphorylation of Thr202/Tyr204-ERK1/2, Ser19-MLC and Thr389-p70S6K, respectively (Fig. 4C and Supplementary Fig. S5A online).

The sustained activation of the MEK1/2-ERK1/2 signaling pathway may also have a negative role on the proliferation of hepatocytes. Indeed, over-activation of the cascade inhibits cell replication of rat hepatocytes and human transformed hepatic cells^2,44^. Therefore, following the two waves of proliferation, we transitorily inhibited the MEK1/2-ERK1/2 pathway for 48 h with the inhibitor U0126 (Fig. 4D and Supplementary Fig. S5B online) and then quantified the expression of proliferation markers Cyclin D1 and KI67 in spheroids on the following days (Fig. 4E,F). The cumulative indexes indicated that 50% to 70% of the PHH could undergo a new cell cycle after transient MEK1/2-ERK1/2 signaling pathway inhibition. No positive cells could be detected in the solvent control experiments (Fig. 4E) or in 2D cultures after the same transient inhibition of the MEK1/2-ERK1/2 signaling pathway (result not shown).

### 3D cultured PHH as hepoid are characterized by long-term differentiation and optimal drug detoxification capabilities

**Figure 5:**
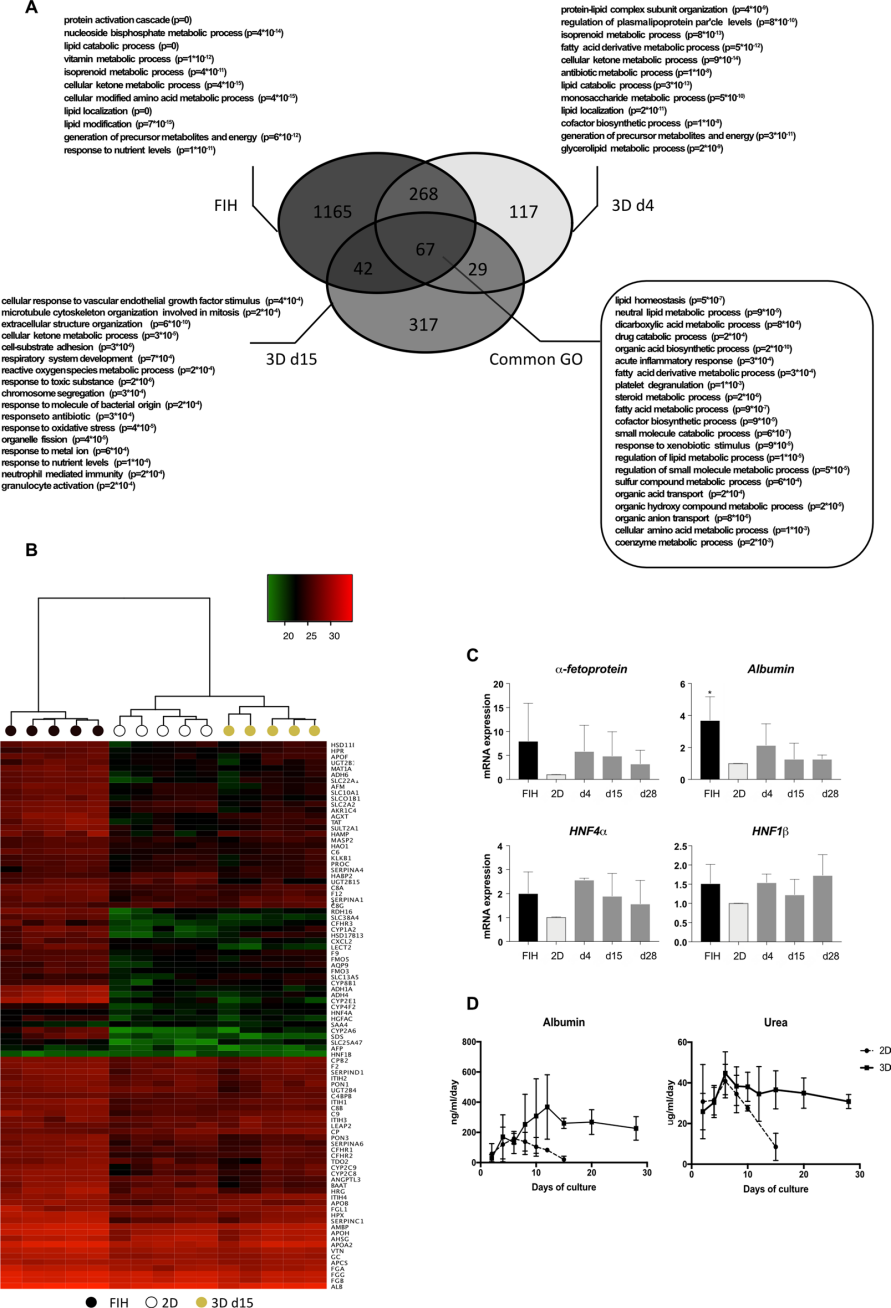
Transcriptomic analysis reveals great similarities between FIH and 3D cultured PHH. (**A**) Transcriptomic analysis of up-regulated genes in FIH and 3D cultured PHH cultured during 4 (d4) and 15 (d15) days. The numbers indicate the numbers of differentially up-regulated genes compared to 2D culture with fold change > 2 and p < 0.05 using WebGestalT. In bold, the most enriched Go Terms found in Webgestald online tool, an important part of them being shared between the three conditions (Common GO). Indicated p-values are after Benjamini–Hochberg multiple testing correction. (**B**) Heatmap showing levels of major hepatic genes (LiGEP) in FIH (black), at maximum differentiation in 2D culture at d4 (white) and in 3D cultured PHH at d15 (yellow). (**C**) Quantification of the gene expression patterns by RT-qPCR of *α-fetoprotein*, *albumin*, *HNF4α* and *HNF1β* in FIH (black), 2D cultures (light grey) and 3D cultured PHH (dark grey), 4 (d4), 15 (d15) and 28 days (d28) after seeding. The results are from at least three independant experiments and are expressed according to the 2D culture level (Mean ± SD, *p < 0.05, one-way ANOVA test). (**D**) Albumin and urea secretions in PHH cultured in 2D or in 3D at the indicated times of culture. The results are from at least three independant experiments (Mean ± SD).

By transcriptomic analysis, we compared RNA expressions of FIH and 3D cultured PHH at days 4 and 15 to 2D cultures, condition considered as the gold standard for PHH. Compared to 2D cultures, 481 and 455 genes were up-regulated in 3D cultured PHH at day 4 and 15, respectively (Fig. 5A), in which 335 and 109 genes were found also up-regulated in FIH (freshly isolated human hepatocytes). Analysis of the enrichment of genes sets has shown that common up-regulated functions (see go-term in box, Fig. 5A) are related to major liver functions such as lipid metabolism and homeostasis, fatty acid process, drug catabolic process and response to xenobiotic stimulus, dicarboxylic acid metabolic and organic acid biosynthesis processes. We also analyzed the differentially expressed biological functions between 3D cultured PHH at day 15 and 2D cultures. Among the up-regulated functions in Hepoid, some are particularly linked to cell proliferation: microtubule cytoskeleton organization involved in mitosis, chromosome segregation, organelle fission. In 2D cultures, pro-inflammatory genes were up-regulated, as well as genes related to the response to chemokines, cellular chemotaxis, inflammatory responses, external stimulus responses and cytokines, which may indicate that 2D cultured PHH were under greater stress. On the contrary, genes related to xenobiotic metabolism enzymes (CYPs, phase II enzymes and transporters) were up regulated in 3D cultured PHH (enrichment ratio greater than 2) (see Supplementary Fig. S6 online). Moreover, we examined the expression of genes previously identified as markers for ductal liver, stem cells, hepatoblasts and mesenchymal cells (see Supplementary Fig. S7A online). Our results showed that most of the genes analyzed (65/78) were identically expressed in 3D cultures at different times of culture and with respect to FIH and 2D cultures. Associated with the absence of detection of the EpCAM protein in 3D cultures (see Supplementary Fig. S7B online), our results showed that expression of stem cell and hepatoblast markers is undetectable in PHH cultured in 3D.

Then, we looked at the expression of specific liver genes, listed for most of them in the LiGEP panel defined by Kim et al.^45^. This panel is based on the significantly differential RNA expression between liver and non-liver samples. They developed an algorithm based on RNA-sequencing analysis to assess the differentiation or maturation status of 93 liver-specific genes. The LiGEP heatmap revealed a strong homology of gene expression profiles between the 2D cultures at day 4 and 3D cultured PHH at day 15 (Fig. 5B). Interestingly, we showed good maintenance of many LiGEP gene expression between FIH and 3D cultured PHH after 2 weeks in the hydrogel. At day 28, the expression of most LiGEP genes decreased slightly to a level close to that of 2D cultures at day 4 while some were still expressed at a high level, close to FIH level. To confirm the role of 3D culture on spheroids differentiation, we looked at the expression levels of several liver-specific genes such as *albumin*, *α-fetoprotein*, *HNF4*α and *HNF1β* by RT-qPCR. It is interesting to note that the level of all these mRNA in spheroids were close to those detected in FIH and also maintained for at least 28 days of culture (Fig. 5C). Expressions of these mRNA were higher than in 2D cultured cells. Then, we showed that 3D cultured PHH were able to secrete albumin and urea at constant levels for at least 4 weeks, unlike 2D cultures during which a sharp decrease was observed after 10 days (Fig. 5D).

**Figure 6:**
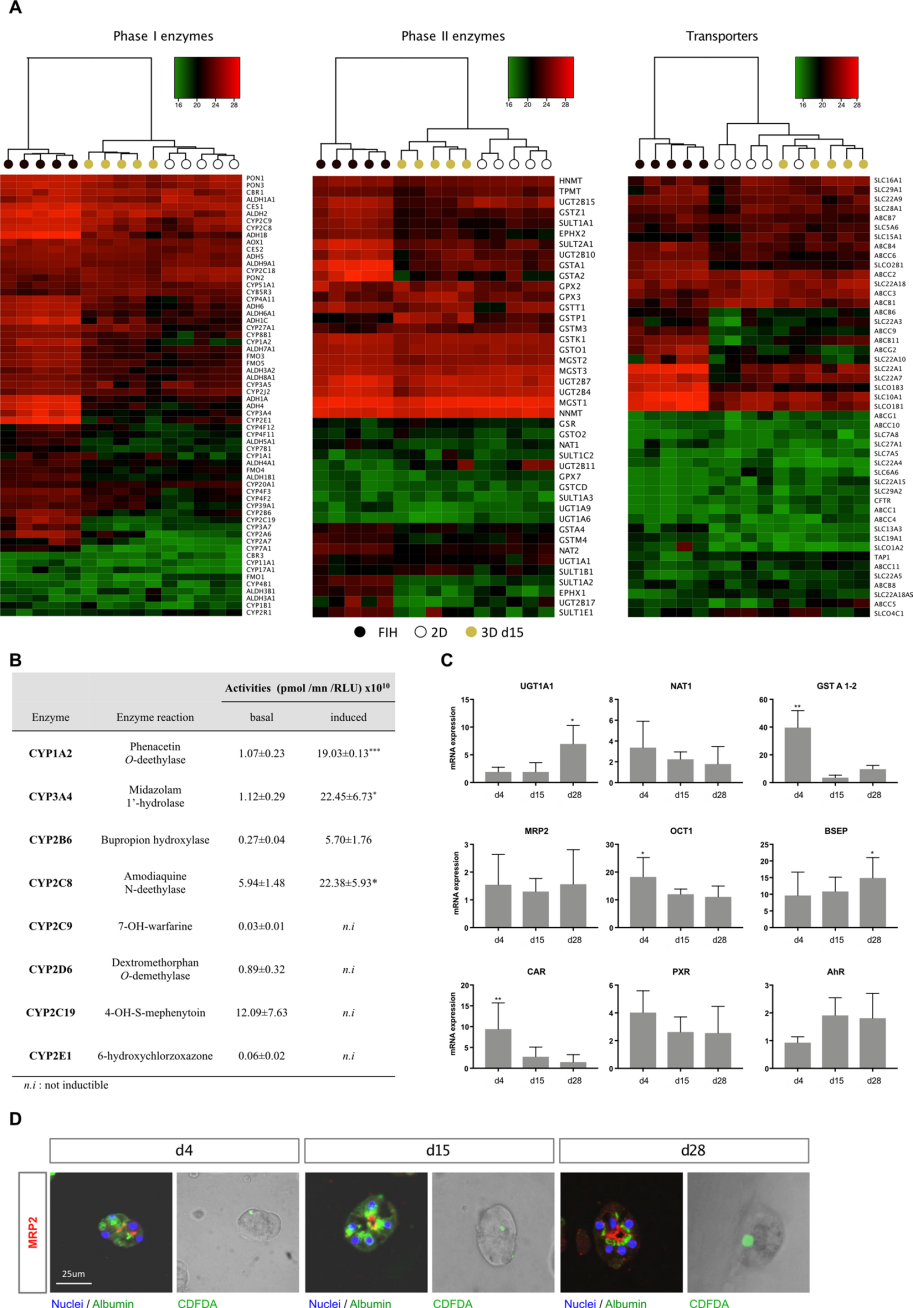
3D cultured PHH exhibit optimal drug detoxification capabilities. (**A**) Heatmap showing the expression of the main phase I and II enzymes and drug transporters in FIH (black), 2D cultures (white) and in PHH cultured in 3D at d15 (yellow). (**B**) LC/MS–MS assays of the activities of the major CYPs isoforms involved in the liver detoxication of xenobiotics in 3D cultured PHH at d15. CYP1A2 was induced by 3-methylcholantrene (5 μm, 24 h), CYP3A4 and CYP2C8 were induced by rifampicin (50 μM, 72 h) and CYP2B6 was induced by phenobarbital (3.2 mM, 72 h) (Mean ± SD, *p < 0.05, **p < 0.01, ***p < 0.001, Student’s t-test). (**C**) Quantification of mRNA levels of phase II (*UGT1A1*, *NAT1*, *GSTA1/2*), hepatic transporters (*MRP2*, *OCT1*, *BSEP*) and CYPs regulators (*CAR*, *PXR*, *AhR*) in PHH cultured in 3D at d4, d15 and d28. The results are from at least three independant experiments and are expressed according to the 2D culture level (Mean ± SD, *p < 0.05, one-way ANOVA test). (**D**) Immunostaining and functional analysis of the MRP2 drug transporter in spheroids revealed bile canaliculi from the beginning to the late stages of the culture (d4, d15 and d28). MRP2 (red), albumin (green) and nuclei (blue) are detected by immunostaining. Functionality of the transporter was assessed by CDFDA processing (green). Scale bar = 25 μm.

Transcriptomic analysis of phase I and II biotransformation enzymes and transporters revealed a very high similarity in gene expression profiles between 3D cultured PHH at day 15, 2D cultures and FIH, thus validating the good maintenance xenobiotic metabolism functions of Hepoid (Fig. 6A). This was confirmed at the functional level by using several specific substrates. Thereby, analysis by LC–MS of CYP functional activities implicated in drug metabolism process in 3D cultured PHH at day 15 demonstrated the ability of major CYPs to be functional at basal level and inducible after treatment with typical inducers (Fig. 6B). Indeed, liver-specific CYP1A2 activity was detected at the basal level and was significantly increased by 3-methylcholanthrene (17-fold). Basal activities of CYP3A4, 2B6, 2C8, 2C9, 2D6, 2C19 and 2E1 were also detected. CYP2B6 could be induced by phenobarbital (20-fold) and CYP2C8 and CYP3A4 by rifampicin (21 and 4-fold, respectively). This potentiation highlights the high expression of biotransformation enzymes and the preservation of a very powerful detoxification capability of Hepoid. Basal and induced EROD (CYP1A, 1B) and MROD (CYP1A2) activities (see Supplementary Fig. S8 online) were higher in 3D cultured PHH than in 2D cultures whatever the time of culture considered, thus confirming the good maintenance of xenobiotic detoxification functions over time and their ability to be regulated. In particular, the activity of CYP1 is about 20% in 2D compared to Hepoid. According to previously published data^46^, we therefore concluded that the activity of CYP1 in the Hepoid is closed to that of FIH during at least 28 days, whatever the time of culture considered. Similarly, we showed by RT-qPCR that phase II xenobiotic metabolism enzymes (*UGT1A1*, *NAT2*, *GSTA1/2*), transporters (*MRP2*, *OCT1*, *BSEP*) and transcription factors (*CAR*, *PXR*, *AhR*) involved in the regulation of detoxifying pathways were also strongly expressed in 3D cultured PHH compared to 2D cultures (Fig. 6C). Moreover, the phase III xenobiotic metabolism protein MRP2 was found exclusively on the apical/bile canalicular domain, thus highlighting the proper polarization of the cells in spheroids. Functional activity of MRP2 confirmed a clear efflux in the central lumen of the spheroids at days 4, 15 and 28 (Fig. 6D). All these data indicate that the differentiation of Hepoid is significantly increased compare to PHH in 2D culture conditions and close to hepatocytes in situ.

## Discussion

The liver has unique regenerative capacities after acute injuries or partial hepatectomy—it recovers its functional mass within a few weeks in human—but so far, no human hepatocyte in vitro has ever shown proliferative activity summarizing the regenerating process. We show here that highly differentiated and polarized human hepatocytes in a 3D collagen matrix, named Hepoid, can undergo multiple divisions for which we characterized two waves of proliferation. This proliferative human hepatocyte capacity, in association with the histological normal characterization of our human cells, opens new fields of very innovative applications in genotoxicology and biotechnology.

The inability to expand human hepatocytes has led to the emergence of alternative human models such as hepatocytes transfected by HPV or transdifferentiated reprogramming fibroblasts^15,19,47^. In these models, cells show a high rate of proliferation but do not yet reach the differentiation level of the mature phenotype compared to adult PHH whereas human hepatic progenitor cells appear to be more related to mature hepatocytes as recently described^32^. Interestingly, Huch et al. described conditions allowing from human liver, long-term expansion of adult bile duct-derived bipotent progenitor cells able to re-differentiate into mature hepatocytes^22^. By this protocol, Garnier et al. proposed to amplify human hepatocytes as 3D organoids, without the need of sorting EpCAM positive cells^23^. In Hepoid, the heatmap of most liver-specific genes remained at levels closed to FIH and all cells were positive for Albumin, MRP2, N- and E-cadherin while most of the genes identified as markers for ductal liver cells, stem cells, hepatoblasts and mesenchymal cells as well as the EpCAM protein were not or only poorly expressed, thus demonstrating the homogeneous mature phenotype of the PHH. Thereby, polarized PHH survived for at least 4 weeks in a well-differentiated state, especially regarding detoxification functions, xenobiotic metabolism enzymes of phase I and II as well as transporters without any apoptosis detection. By ensuring long-term survival and concomitant differentiation, Hepoid allows, while taking into account of inter-individual variability, to perform acute and chronic toxico-pharmacological studies necessary to overcome inter-species specificities and biased interpretations that could be made with animal models, both in vitro and in vivo. Hepoid remained phenotypically stable and retained specific hepatic functions in accordance with the cholestatic, steatogenic and chronic toxicity studies performed on others PHH 3D models^25,26,48–50^.

Herein, our results demonstrated that in an optimized and well-defined 3D culture model, PHH could undergo two waves of proliferation during the two first weeks of culture. The establishment of cell–cell interactions by a transient LAP culture prior to incorporation into the collagen I matrix is crucial for the formation and proliferation of PHH spheroids. We postulate that almost all human hepatocytes cultured as Hepoid undergo at least two cell cycles during the two first weeks of culture since a very high level of nuclei appeared positive for cell cycle markers (Cyclin D1/KI67, BrdU/EdU, phospho-Histone H3). As an example, the mean cumulative index of the phase M markers reached 230% and 150%, during the first 2 weeks of culture, respectively. Subsequently, no more positive nucleus was detected in the Hepoid, as in the population of isolated PHH. The limited growth rate of human hepatocytes in vitro in Hepoid is consistent with the limited proliferation of rodent hepatocytes in vivo after partial hepatectomy and in vitro (one or two cycles)^1,51^. Interestingly, long-term expansion of proliferating mouse and human hepatocytes as organoids which recapitulate hepatocyte proliferation upon partial-hepatectomy has been recently reported^21^. In this model, organoids from pediatric and adult primary hepatocytes appeared to be more limited in their expansion times when compared to the fetal cultures.

The proliferation of PHH cultured as Hepoid can be obtained in the absence of FBS and growth factors (EGF, HGF, ITS) as the removal of these factors only partially decreases the rate of proliferation, up to 41% for FBS. The proliferation of primary mouse hepatocytes is also known to occur in vitro in the absence of growth factors and FBS^52^ while HGF, EGF and TGFα have been shown to be the major mitogens necessary for late G1 progression and G1/S phase transition in rat hepatocytes^3,53–55^. It has been recently shown that when cultured in 3D Matrigel, primary mouse hepatocytes form few colonies and decline rapidly when cultured in the presence of EGF and HGF^56^. Besides, Hu et al. obtained using an optimized complex medium, small organoids from murine and human fetal hepatocytes that expand to a diameter of 400 microns in 15–20 days and could be passaged by mechanical disruption^21^. Organoids from pediatric and adult primary human hepatocytes appeared to have more limited expansion times than those from fetal hepatocytes. In our work, we clearly demonstrate that the proliferation of Hepoid from adult hepatocytes depends on both cell–cell interactions and the 3D microenvironment provided by the matrix. A precise window of collagen concentration, 0.75–3 mg/ml, was determined for an optimal proliferation. At the concentration of 4 mg/ml, proliferation was reduced by 82%, indicating that higher matrix rigidity is responsible for blocking adult human hepatocyte proliferation. At lower stiffness (less than 3 mg/ml), embedment in the 3D matrix forces the proliferating cells to adopt a spheroid structure. Over the time of culture, the collagen fibers concentrate in the microenvironment close to the cells, thus progressively increasing the local rigidity, which could be responsible for the inhibition of cell proliferation. In our model, the enhancement of cell–cell interactions, through transient culture in LAP, is also fundamental for untransformed adult human hepatocytes that are poorly motile cells that therefore cannot easily establish cell–cell communications, essential for the development and growth of spheroids. Without improved cellular interactions, the proliferation decreased by 83%. Besides, in both 3D-LAP and 2D cultures, PHH failed to proliferate, highlighting that cell–cell interactions and the 3D microenvironment in the collagen matrix are key factors in the initiation of the human hepatocyte cell cycle.

Furthermore, hepatocytes embedded into the collagen matrix according to our method can reinitiate a new cycle after transient inhibition of the MEK1/2-ERK1/2 signaling pathway. The MAPK MER1/2-ERK1/2 pathway has been demonstrated as a major player of normal hepatocytes proliferation in rodents and transformed human hepatic cells. On the contrary, sustained activation of this pathway induces growth arrest in different tumor cellular models including human hepatocarcinoma cells and rat hepatocytes in primary culture^2,57^. Over-expression of an active form of MEK1/2 is sufficient to block hepatoma cells proliferation^44^. In this context, we demonstrated that the blocking of PHH proliferation capabilities in vitro after the first two waves could be supported by the sustained activity of MAPK. Herein, transient inhibition of the MEK cascade can re-potentiate the PHH to perform a new wave of replication allowing the study of the major regulators or disruptors that may be involved in the regulation or the disturbance of the human hepatocyte cell cycle. The present study is a first step towards achieving a higher rate of human hepatocyte proliferation. Further stimulations of the cell cycle by cytokines or growth factors as well as possible passages of Hepoid without loss of differentiated functions are ongoing for obtaining additional waves of proliferation. During the review process of the manuscript, it was reported that activation of the Wnt signaling is sufficient to drive the proliferation of at least 50% of the PHH cultured in LAP^58^.

Importantly, Hepoid makes it possible to consider acute and chronic genotoxicity-mutagenicity studies in normal proliferating human hepatocytes. It is of particular interest to understand the mutations found in Hepatocellular Carcinoma (HCC) tumors, a major cause of death in industrialized countries^59^, and improved the public information regarding the involvement of new environmental contaminants in the occurrence of HCC^60^. Thereby, potentially genotoxic environmental compounds and pharmacological drugs could be analyzed using the Hepoid model to assess the mutations spectra that occur in normal proliferating hepatocytes. In human liver, most hepatocytes are considered quiescent with a replacement rate of 300–500 days. Thus, considering that about 2–3 cells/1000 proliferate each day, the effects of genotoxic drugs on normal proliferating cells represents a major issue. Our approach will provide data on potential carcinogenic compounds in PHH to determine specific mutational fingerprints.

Altogether, we generated in vitro proliferating and long-term differentiated human adult normal hepatocytes with functional epithelial polarization, allowing acute and chronic pharmacological, toxicological and mutagenesis studies in well-differentiated PHH for at least 4 weeks. In addition, Hepoid offers, for the first time, the possibility to study in-depth the mechanisms regulating the initiation, progression and re-initiation phases of the primary human hepatocyte cell cycle. This is an essential and expected central step, both to understand the mechanism that regulates the cell cycle of human hepatocytes and ultimately to obtain a source of normal human hepatocytes necessary and expected by many laboratories for basic research and biotechnological applications.

## Material and methods

### Cell culture

#### Cell isolation and 2D culture

Human liver samples were obtained from patients undergoing liver resection through the Centre de Ressources Biologiques (CRB) Santé of Rennes (CHRU Pontchaillou, Rennes, France, http://www.crbsante-rennes.com) and approved by the Inserm Ethical Review Committee (October 8, 2013-IRB00003888) (Table 1). Freshly isolated human hepatocytes (FIH) were obtained from the non-tumoral part of the biopsy, certified histologically normal and isolated by a two-step collagenase perfusion procedure as described previously^61^. Cryopreserved PHH were obtained from Biopredic International (Rennes, France). The research protocol was conducted under French legal guidelines and the local institutional ethics committee. Written informed consent was obtained from all donors of liver material. For 2D cultures, cells were seeded in MW96 plates at a density of 4.5 × 10^4^ cells/well in William’s medium supplemented with bovine serum albumin (1 g/l), glutamine (2 mM), bovine insulin (5 μg/ml), penicillin (100 U/ml), streptomycin (100 μg/ml) and fetal bovine serum (FBS) (10% v/v). The culture medium was renewed with hydrocortisone hemisuccinate 54 μM without FBS. 2D cultures were stopped after 4 days.

#### Collagen 3D culture

In order to promote the establishment of cell–cell interactions and the formation of cell clumps which will then be embedded into the collagen matrix, fresh or cryopreserved hepatocytes were first incubated overnight (i.e. 12–15 h) in ultra low-attachment plate (LAP) (Corning Costar) at the concentration of 2 × 10^6^ cells/well of a MW6 plate in the medium described previously before being embedded in the collagen matrix. Collagen type I (Sigma-Aldrich) was diluted into the culture medium and the pH was adjusted at 7.4 before the cells were added at a concentration of 3.5 × 10^5^ cells/ml. This mix of cells and collagen was poured into 96-well plates (100 μl) or 48-well plates (250 μl) and incubated at 37 °C, 5% CO_2_. After polymerization, an equal volume of medium was added. Cells were maintained in the medium described previously supplemented with hydrocortisone hemisuccinate 1.08 μM, ITS (Recombinant insulin 10 μg/ml, transferrin 5.5 μg/ml, sodium selenite 5 ng/ml) (Sigma-Aldrich), rhHGF (2.5 ng/ml) (BioLegend) and rhEGF (50 ng/ml) (PeproTech). The medium was renewed every 48–72 h. This method is protected under an international patent (EP2018030560320180516/WO2019219828)^62^.

#### Collagen gels inclusion in paraffin

After fixation in formol 4%, collagen gels were dehydrated with successive baths of alcohol and xylene before being impregnated with paraffin using EXCELSIOR ES tools (Thermo Scientific). After impregnation, gels were included in paraffin blocs and 4 μm cuts were made.

#### HUCCT1 cells culture

HUCCT1 cells were thawed and cultured in 2D according to the supplier’s recommendations (Riken BioResource Center, Japan). Once they reached confluence, cells were directly embedded in 1.5 mg/ml collagen gels as described previously for PHH and were cultured for 8 days in the appropriate medium.

### TPEF microscopy

Two-photon excitation fluorescence (TPEF) microscopy imaging was performed on mRIC facility of Biosit, University of Rennes1 (France). The imaging system composed of a confocal TCS SP5 scanning head (Leica Microsystems, Mannheim, Germany) was mounted on a inverted microscope Leica DMSI 6000 CS (Leica Microsystems) and equipped with a MAITAI femtosecond laser (Spectra Physics, Santa Clara, CA). A 10× dry objective (NA = 0.4; Leica Microsystems), a 20× oil immersion objective (NA = 0.7; Leica Microsystems) and 60× water immersion objective (Olympus LUMFL 60 W × 1.1NA) were used. Image processing was performed using ImageJ software (National Institutes of Health; http://rsb.info.nih.gov.gate2.inist.fr/ij/).

### Quantification of DNA synthesis

#### Incorporation of thymidine analogues

After 24 h of incorporation, BrdU positive cells were detected using the anti-BrdU antibody (GE Healthcare). 5-Ethynyl-2′-deoxyuridine EdU (10 μM) (Thermo Fisher), another thymidine analogue, was also used to quantify DNA synthesis. After 24 h of incorporation, cells in collagen gels were fixed in formol 4% and included in paraffin before EdU was revealed by CY5-azide (10 μM) (Sigma-Aldrich).

#### Mitotic index

Cells were treated with colcemid (1 μM, 24 h) to halt cells in the M phase before being fixed in formol 4%. The quantification of the mitotic index was assessed by immunostaining of the phosphorylated histone H3.

#### Blockage of hepatocyte proliferation

From the moment of seeding in LAP, cells are treated with the MEK inhibitor U0126 (20 μM) (Promega), the MLCK inhibitor ML7 (20 μM) (Santa-Cruz Biotechnology), the PI3K/mTOR inhibitor rapamycin (10 nM) (Merck) or with vehicle (DMSO 0.1%). Cells were then embedded in collagen as described previously using medium supplemented with inhibitor or vehicle. Medium was renewed twice a day for 6 days.

### Biochemical assays

#### Assessment of viability

Cell viability was assessed by determining the ATP content of PHH cultured in 3D with the CellTiter-Glo 3D Luminescent Cell Viability Assay (Promega).

#### Albumin and urea secretion

Cell culture medium was collected every 48 h for the dosage of albumin and urea content. Albumin concentration was determined using the Human Serum Albumin DuoSet ELISA kit (R&D Systems) according to the manufacturer’s guidelines. Urea secretion was determined using a ChromaDazzle Urea Assay (Assay Genie) according to the manufacturer’s protocol.

#### Immunohistochemistry

Immunohistochemical staining was performed on the Discovery Automated IHC stainer using the Discovery Rhodamin kit (Ventana Medical Systems). Following deparaffination with Ventana EZ Prep solution at 75 °C for 8 min, antigen retrieval was performed using Tris-based buffer solution CC1 (Ventana Medical Systems) at 95–100 °C for 36 min. Endogen peroxidase was blocked with 3% H_2_O_2_ for 8 min at 37 °C. After rinsing with reaction buffer, slides were incubated at 37 °C for 60 min with an appropriate dilution of the following primary antibodies: KI67 (SP6, 1:400, Thermo Fisher) and EpCAM (1B7, 1:100, Thermo Fisher), phospho-Histone H3 (Ser10) (06-570, 1:100, Merck), E-cadherin (24E10, 1:100, Cell Signaling) and cleaved-caspase 3 (#9661, 1:100, Cell Signaling), N-cadherin (32/N, 1:100, BD Biosciences), MRP2 (M2 III-6, 1:100, Abcam), MKL1 (ab113264, 1:100, Abcam) and Cyclin D1 (SP4, 1:100, Abcam). After rinsing, signal enhancement was performed using the Ventana Rhodamin kit and an HRP secondary antibody HRP (Roche) was incubated for 16 min. After removal from the instrument, slides were manually rinsed, stained with Albumin (A80-229A, 1:100, Ozyme), secondary antibody for albumin detection (Donkey anti-goat 647, 1:250) and DAPI (1:1500) and cover slipped. The labeled nuclei were quantified using a fluorescence Eclipse Ni-E microscope (Nikon) equipped with a photonic camera Orca R2 (Hamamatsu).

#### Immunoblotting analysis

Cells were extracted from collagen gels by the action of Liberase TL (Roche) for 1 h at 37 °C. After having been lysed and dosed, protein samples were separated using SDS-PAGE and transferred onto nitrocellulose membranes in a transfer buffer (25 mM Tris, 200 mM glycine, Ethanol 20%). The blots were blocked with 5% low-fat milk in Tris-buffer saline (65 mM Tris pH 7.4, 150 mM NaCl) at room temperature for 1 h. They were incubated overnight with primary antibodies at 4 °C: p-p44/42 MAPK (Thr202/Tyr204) (E10, 1:1000), p-MLC Ser19 (#3671, 1:500), p-P70S6K Thr389 (1A5, 1:500, Cell signaling), β-actin (AC-15, 1:1000, Sigma-Aldrich), HSC-70 antibody (B-6, 1:5000, Santa Cruz Biotechnology). After being washed with TBS, the blots were incubated for 1 h with an HRP secondary antibody (1:1000) in 5% low-fat milk in TBS at room temperature. The blots were then washed with TBS. Immunocomplexes were visualized with a ChemDoc MP Imaging System (Bio-Rad) after a chemiluminescent reaction using the Ommobilon Western Chemiluminescent HRP substrate (Merck Millipore).

#### CYPs enzyme activities measurement by LC–MS

After 15 days of culture, Hepoid were treated with 3-methylcholantrene (5 μM, 24 h) (Sigma-Aldrich) for CYP1A2 induction, rifampicin (50 μM, 72 h) (Sigma-Aldrich) for CYP3A4 and CYP2C8 induction, phenobarbital (3.2 mM, 72 h) for CYP2B6 induction and isoniazide for CYP2E1 induction. For basal activities, cells were treated with vehicle (DMSO, 0.1%). Cells were incubated in DMEM containing usual specific probe substrates, i.e. phenacetin (CYP1A2, 200 μM), midazolam (CYP3A4, 50 μM), bupropion (CYP2B6, 100 μM), amodiaquine (CYP2C8, 100 μM), warfarin (CYP2C9, 100 μM), dextromethorphan (CYP2D6, 100 μM), S-mephenytoin (CYP2C19, 100 μM) and chlorzoxazone (CYP2E1, 100 μM). Supernatants were collected after 4-h incubation. Formed metabolites (acetaminophen, 1-OH-midazolam, OH-Bupropion, desethyl-amodiaquine, 7-OH-warfarin, dextrorphan, S-mephenytoin 4′-hydrolase and 6-OH chlorzoxazone, respectively) were quantified by liquid chromatography-tandem mass spectroscopy (LC/MS–MS), using an high-performance liquid chromatography Aria system (Agilent), equipped with a Poroshell 120 C18 (2.7 × 100 mm) column (Agilent Technologies) and coupled to a tandem mass spectrometry TSQ Quantum Ultra (Thermo Fisher Scientific) fitted with an electrospray ionization source (ESI+). For all analyses, HPLC gradient consisted in a mobile phase starting with 10% of organic phase (acetonitrile/methanol, 50/50, v/v) and 90% of water with 0.1% of formic acid, shifting to 95% of organic phase and using a linear gradient (flow rate of 0.5 ml/min). Acquired data were processed with Xcalibur software (Thermo Fisher Scientific). A CellTiter Glo-3D assay (Promega) was then performed to normalize activity with the number of viable cells.

#### CYPs activities measurement by spectrofluorimetry

Ethoxyresorufin O-deethylation (EROD) and Methoxyresorufin O-deethylation (MROD) associated with CYP1A1/2-1B1 and CYP1A2 activities, respectively, were measured as described by Burke and Mayer^63^. Briefly, cells were washed with PBS at 37 °C before being incubated with salicylamide (1.5 mM) to block phase II-conjugation enzymes. 7-Ethoxyresorufine or 7-methoxyresorufine (Sigma-Aldrich) were added 1 min later and the oxidation of these substrates was measured by fluorescence detection every 2 min during 20 min at 37 °C. The reaction rate was linear with time. A CellTiter-Glo 3D assay (Promega) was then performed to normalize activity with the number of viable cells.

### MRP2 transporter activity

3D cultures were incubated 30 min with 5(6)-Carboxy-2′,7′-dichlorofluorescein diacetate CDFDA (10 μM) (Sigma-Aldrich), washed twice with PBS and incubated in serum-free medium for 120 min. The fluorescence (Ex.: 470 nm, Em.: 529 nm) was assessed using a fluorescence Eclipse Ni-E microscope (Nikon) equipped with a photonic camera Orca R2 (Hamamatsu).

### mRNA quantification

#### mRNA isolation

Cells were removed from collagen gels by the action of Liberase TL (10 μg/ml) for 1 h at 37 °C. Then, total RNA were extracted from cells pellets using NucleoSpin RNA (Macherey–Nagel), quantified using a NanoDrop ND-1000 (Thermo Fischer) and then used for cDNA synthesis using the High Capacity cDNA reverse transcription kit (Applied Biosystems).

#### RT-qPCR analysis

Real-time PCR for all genes was performed using SYBR green technology (Applied Biosystems) and the CFX384 Real-Time System (Bio-Rad) according to the manufacturer’s recommendations. All the primers used are listed in Table 2. The amplification curves were analyzed with the Bio-Rad CFX Manager software using the comparative regression method. GAPDH was used for the normalization of expression data. The relative amount of measured mRNA in samples was determined using the 2^−ΔΔCT^ method where ΔΔCT = (CT_target_ − CT_GAPDH_)_sample_ − (CT_target_ − CT_GAPDH_)_calibrator_. Final results were expressed as the *n*-fold difference of target gene expression in tested samples compared to the mean expression value of the 2D cultures used as calibrator.

**Table 2:** Primer sequences.

Gene	Forward primer	Reverse primer
*GAPDH*	GTGGACCTGACCTGCCGTCT	GGAGGAGTGGGTGTCGCTGT
*E-CADHERIN*	TGCTCTTGCTGTTTCTTCGG	TGCCCCATTCGTTCAAGTAG
*N-CADHERIN*	GTGCATGAAGGACAGCCTCT	ATGCCATCTTCATCCACCTT
*a-FETOPROTEIN*	TGCAGCCAAAGTGAAGAGGGAAGA	CATAGCGAGCAGCCCAAAGAAGAA
*ALBUMIN*	GGGCATGTTTTTGTATGAAT	CCCACTTTTCCTAGGTTTCT
*HNF4a*	CAGAAGGCACCAACCTCAAC	CTCGAGGCACCGTAGTGTTT
*HNF1β*	GCCTTAGTGGAGGAATGCAA	GAGGGTTCAGGCTGTGAGTC
*UGT1A1*	TGACGCCTCGTTGTACATCAG	CCTCCCTTTGGAATGGCAC
*NAT1*	ACCATTGATGGCAGGAACTA	TGTTCCCTTCTGATTTGGTC
*GSTA1/2*	CGCTACTTCCCTGCCTTTGA	AGTCAAGCTCCTCGACGTAG
*MRP2*	TGAGCAAGTTTGAAACGCACAT	AGCTCTTCTCCTGCCGTCTCT
*OCT1*	TAATGGACCACATCGCTCAA	AGCCCCTGATAGAGCACAGA
*BSEP*	TGATCCTGATCAAGGGAAGG	TGGTTCCTGGGAAACAATTC
*CAR*	TGATCAGCTGCAAGAGGAGA	AGGCCTAGCAACTTCGCATA
*PXR*	CCAGGACATACACCCCTTTG	CTACCTGTGATGCCGAACAA
*AhR*	CTTCCAAGCGGCATAGAGAC	AGTTATCCTGGCCTCCGTTT

*GAPDH* glyceraldehyde 3-phosphate dehydrogenase, *SOX* SRY-box, *UGT* UDP-glycosyltransferase, *NAT* N-acetyltransferase, *GST* glutathione-S-transferase, *MRP* multidrug resistance-associated protein, *OCT* organic cation transporter, *BSEP* bile salt export pump, *CAR* constitutive androstane receptor, *PXR* pregnant X receptor, *AhR* aryl hydrocarbon receptor.

#### Transcriptomic analysis

mRNA were collected from FIH (n = 5), 2D cultured PHH at day 4 (n = 5) and Hepoid at day 4 (n = 5), 15 (n = 5) and 28 (n = 4). RNA samples were checked for degradation based on the RNA Integrity Number (RIN > 8). The RNA libraries were sequenced using the HiSeq 2500 (Illumina) following the protocol previously described^64^. Complementary RNA synthesis, hybridization and chip scanning were performed at the GenoBIRD platform (Nantes, FR). The analysis of the generated data was performed using R. Go terms enrichment analysis of differentially expressed genes (FC > 2, p < 0.05) was performed using WebGestalT (WEB-based GEneSeT) online analysis tool (Vanderbilt University, The Netherlands).

### Statistical analysis

Unless specified, all experiments were performed in triplicate and representative results were presented where data were expressed as mean ± SD. A two-tailed Student’s t-test or a one-way ANOVA test was used and were carried out using GraphPad Prism v.7.0 (GraphPad Software, La Jolla, CA, USA). A Mann–Whitney U test was carried out using RStudio Team (2019) to compare results obtained by transcriptomic analysis.

## Supplementary Information

Supplementary Information.

## Abbreviations

PHH: Primary human hepatocytes
Hepoid (trademark): Hepatocyte hollow spheroids
FIH: Freshly isolated hepatocytes
LAP: Low-attachment plate
CYP: Cytochrome P450
