# Supplementary material for: Generation of proliferating human adult hepatocytes using optimized 3D culture conditions
Source: Sci Rep. 2021 Jan 12;11:515. doi: 10.1038/s41598-020-80019-4 (PMC7804446; doi:10.1038/s41598-020-80019-4)
Supplement: Supplementary file 1 — Supplementary Information. [file 41598_2020_80019_MOESM1_ESM.pdf]

Generation of proliferating human adult hepatocytes using optimized 3D culture conditions

Sophie Rose, Frédéric Ezan, Marie Cuvellier, Arnaud Bruyère, Vincent Legagneux, Sophie Langouët and Georges Baffet*

Univ Rennes, Inserm, EHESP, Irset (Institut de recherche en santé, environnement et travail) - UMR_S 1085, F-35000 Rennes, France

Correspondence to

Dr. Georges Baffet, Dr Sophie Langouët

E-mail : georges.baffet@univ-rennes1.fr, sophie.langouet@univ-rennes1.fr, Tel : + 33(0)223234806

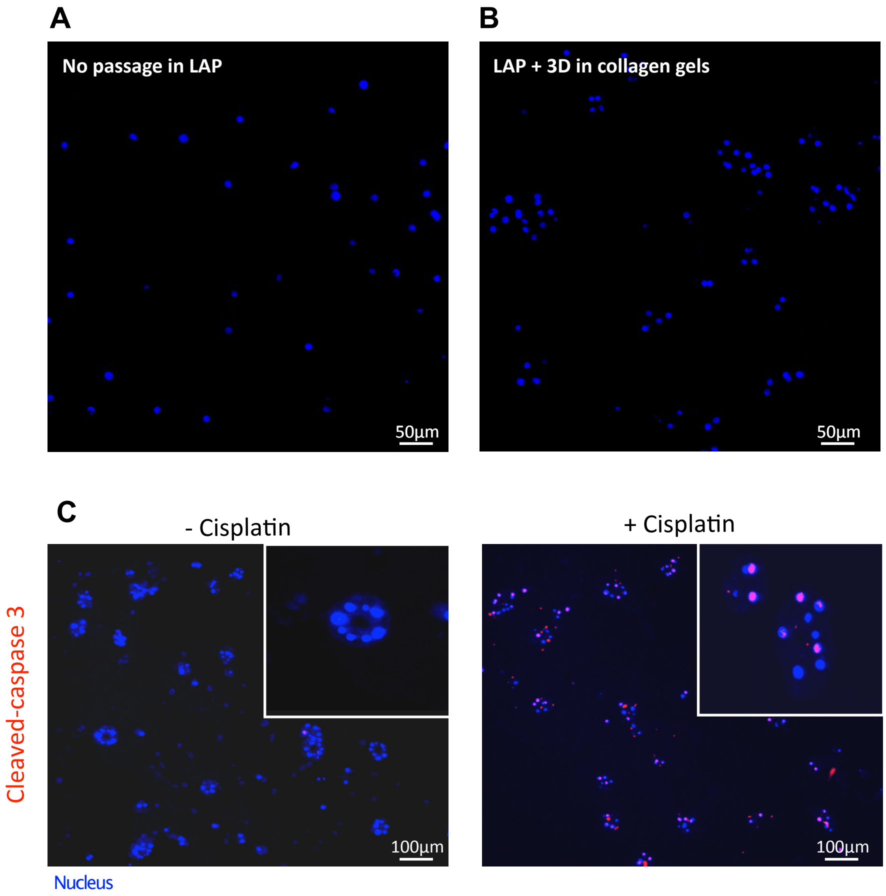

Figure S1. PHH after 6 days of culture in collagen gels. FIH were either (**A**) directly embedded in collagen gels or (**B**) transitorily incubated in low-attachment plate (LAP) prior to inclusion in collagen to promote the establishment of cell-cell interactions. Scale bar = 50 µm. **C.** Cleaved-caspase 3 immunolocalization in Hepoid at day 15 without treatment (-cisplatin) or after a 24h cisplatin treatment (20 µg/ml) (+ cisplatin).

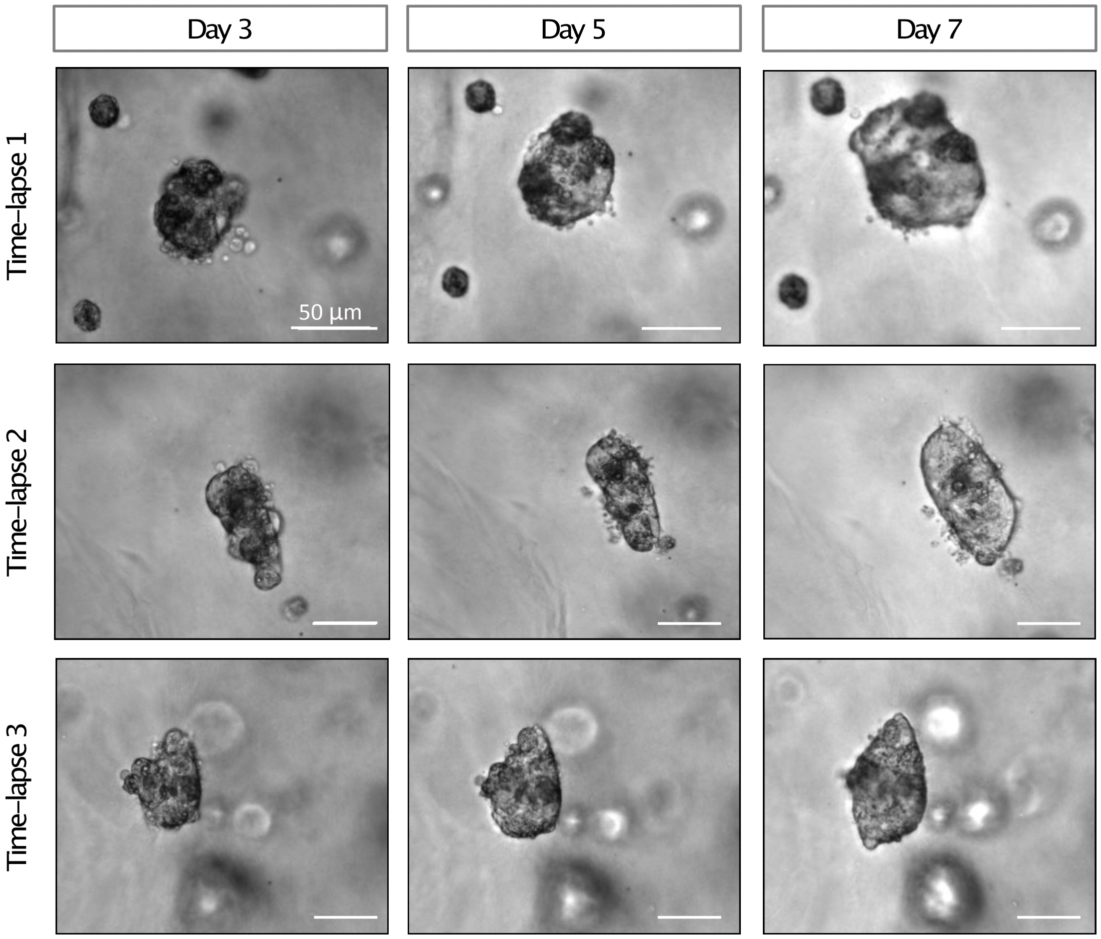

Figure S2. Pictures of proliferating Hepoid at day 3, 5 and 7, extracted from time-lapse videos performed from day 3 to day 7 of culture.

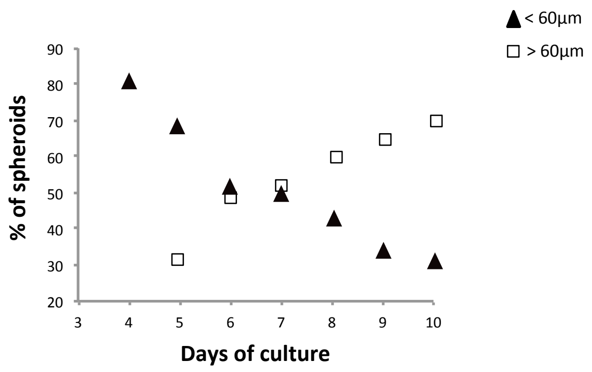

Figure S3. Representation of the decreasing number of spheroids with diameter <60 µm and the increasing number of spheroids with diameter >60 µm as a function of the culture time. Data are from one representative experiment of at least three independent experiments.

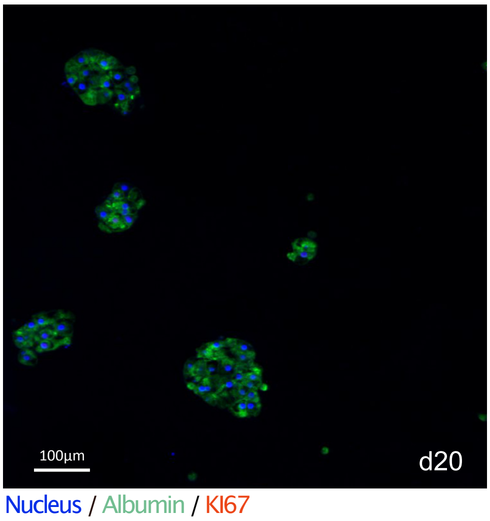

Figure S4. Illustration of the staining of KI67 (red), Albumin (green) and nuclei (blue) in Hepoid at after 20 days of culture. Scale bar = 100 µm.

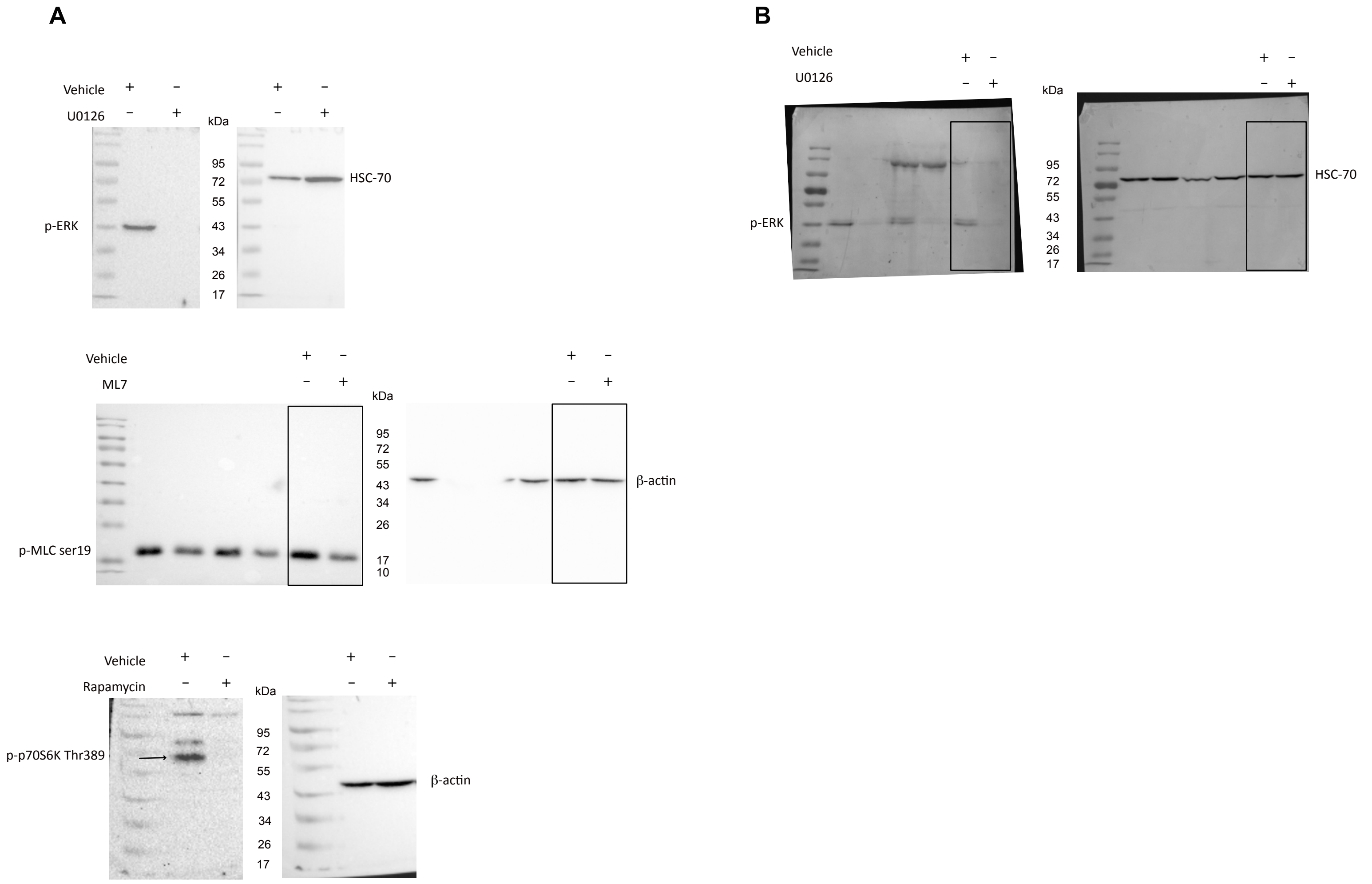

Figure S5. A. Full western-blot images corresponding to those presented in Figure 4C. Blots of interest for phospho-MLC on Serine 19 and the corresponding β--actin are in the selected rectangular area. **B**. Full western-blot images corresponding to those presented in Figure 4D. Blots of interest are in the selected rectangular area.

		Description	Enrichment Ratio	pValue	FDR	Genes
Down-regulated genes in 3D	GO:2000147	positive regulation of cell motility	3,26	1,22E-08	6,40E-06	CCN1;ANXA3;LAMC2;TGFB2;CXCL8;RRAS2;PLK2;PDGFC;SPHK1;ETS1;DEFB1;COL1A1;CAVIN1;CCL7;IQGAP1;GLIPR2;DAPK3;ILK;SRPX2;ITGA2;FLNA;APP;LAMB1;TMSB4X;ICAM1;ADAM9;ACTN4;PGAM4;PHPT1;BMP2
	GO:0031589	cell-substrate adhesion	3,88	1,50E-08	6,40E-06	CCN1;TACSTD2;LGALS1;CCN2;ITGB6;COL1A1;MELTF;IQGAP1;DAPK3;ILK;ITGA2;FLNA;JAG1;DLC1;LAMB1;CD44;CX3CL1;CDKN2A;ADAM9; CD34;ACTN4;VCL;ACTN1;ITGB1
	GO:0032970	regulation of actin filament-based process	3,56	7,82E-08	2,22E-05	TACSTD2;CCN2;TGFB3;PAM;DSC2;ECT2;PDE4D;FRMD6;DAPK3;ILK;CNN2;CAPG;FLNA;DLC1;RHOQ;TMSB4X;ICAM1;CX3CL1;TMSB10;JMY; MYH9;ARHGAP18;NCK1;PHPT1
	GO:0043062	extracellular structure organization	3,35	1,29E-07	2,75E-05	TNC;CCN1;LAMC2;TGFB2;TGFBI;VCAN;LOX;CCN2;ETS1;ITGB6;DPP4;COL1A1;MELTF;CAPG;ITGA2;APP;LAMB1;CD44;ICAM1;RIC8A;DNAJB6; COL6A1;CD34;ITGAE;ITGB1
	GO:0033627	cell adhesion mediated by integrin	8,01	4,18E-07	7,11E-05	TGFB2;TESC;ITGB6;DPP4;LPXN;ITGA2;ICAM1;ADAM9;EPHA2;ITGB1
	GO:0034109	homotypic cell-cell adhesion	6,19	1,41E-05	2,00E-03	LGALS1;DSC2;ILK;FLNA;CD9;CLIC1;MYH9;VCL;ACTN1
	GO:0071216	cellular response to biotic stimulus	3,62	1,83E-05	2,11E-03	CXCL6;CCL2;ANKRD1;CXCL1;CXCL8;VIM;IL18;TLR2;PDE4D;CARD16;RIPK2;ICAM1;CX3CL1;ADAM9;CASP1
	GO:0034612	response to tumor necrosis factor	3,42	1,99E-05	2,11E-03	CCL2;LTB;ANKRD1;CXCL8;SPHK1;COL1A1;CCL7;CARD16;ILK;GBP2;TMSB4X;ICAM1;CX3CL1;ADAM9;ACTN4;CASP1
	GO:0034330	cell junc- on organization	3,20	2,58E-05	2,44E-03	LAMC2;TGFB2;TGFB3;CDH6;ECT2;IQGAP1;DAPK3;ILK;ITGA2;FLNA;CD9;DLC1;FBLIM1;ACTN4;EPHA2;VCL;ACTN1
	GO:0007015	actin filament organization	2,77	4,14E-05	3,52E-03	TACSTD2;CCN2;TGFB3;TPM4;CAPG;FLNA;DLC1;RHOQ;TMSB4X;ICAM1;CX3CL1;TMSB10;JMY;ACTN4;CAP2;ARHGAP18;TNFAIP1;NCK1; ACTN1;ITGB1
	GO:0051493	regulation of cytoskeleton organization	2,47	6,46E-05	4,99E-03	TACSTD2;PLK2;CCN2;TGFB3;PAM;ECT2;MAP2;DAPK3;ILK;CAPG;FLNA;DLC1;RHOQ;TMSB4X;ICAM1;CX3CL1;TMSB10;JMY;ARHGAP18;NCK1;PHPT1;CKAP2;RASSF1
	GO:0007162	negative regulation of cell adhesion	3,13	9,84E-05	6,97E-03	TNC;TACSTD2;TGFBI;LGALS1;COL1A1;MELTF;LPXN;JAG1;CD9;DLC1;CX3CL1;CDKN2A;ACTN4;FXYD5;BMP2
	GO:0007229	integrin-mediated signaling pathway	4,78	1,11E-04	7,29E-03	CCN2;ITGB6;ILK;ITGA2;FLNA;ADAM9;ITGAE;MYH9;ITGB1
	GO:0042476	odontogenesis	4,26	1,24E-04	7,50E-03	TNC;TGFB2;ID3;TGFB3;PAM;TUFT1;COL1A1;LAMB1;CD34;BMP2
	GO:0002576	platelet degranulation	4,22	1,32E-04	7,50E-03	TGFB2;TGFB3;FLNA;APP;CD9;TMSB4X;ACTN4;CD109;VCL;ACTN1
	GO:0002791	regulation of peptide secretion	2,43	1,64E-04	8,71E-03	ANKRD1;TGFB2;IL1RL1;TGFB3;CLTRN;PAM;SOX4;TLR2;DPP4;RHBDF2;CARD16;CRP;PAX8;TMSB4X;RIPK2;CX3CL1;ADAM9;CD34;CASP1; EXOC1;PHPT1
	GO:0036230	granulocyte activation	2,36	1,77E-04	8,83E-03	CXCL6;GLIPR1;ANXA3;CXCL1;CXCL8;IL18;QPCT;ADGRE5;TLR2;COTL1;CPPED1;S100A11;IQGAP1;CNN2;PKM;CD44;CTSZ;DEGS1;RAB24;CD59;VCL;CTSC
	GO:0070482	response to oxygen levels	2,71	2,02E-04	9,55E-03	ANKRD1;TGFB2;PMAIP1;CCN2;TGFB3;PAM;ETS1;TLR2;DPP4;COL1A1;CARD16;ITGA2;PKM;ICAM1;CD34;ACTN4;BMP2
	GO:0034341	response to interferon-gamma	3,35	2,61E-04	1,17E-02	CCL2;VIM;TLR2;CCL7;DAPK3;CAMK2G;GBP2;CD44;ICAM1;CX3CL1;IFNGR1;CASP1
	GO:0090130	migration	2,84	2,82E-04	1,19E-02	ANXA3;TGFB2;TACSTD2;PLK2;ETS1;DPP4;S100A2;GLIPR2;SRPX2;ITGA2;TMSB4X;ADAM9;MYH9;EPHA2;ITGB1
	GO:0001667	ameboidal-type cell migration	2,53	2,95E-04	1,19E-02	ANXA3;TGFB2;TACSTD2;PLK2;ETS1;DPP4;S100A2;GLIPR2;ILK;SRPX2;ITGA2;TMSB4X;ARID5B;RIC8A;ADAM9;MYH9;EPHA2;ITGB1
	GO:0050900	leukocyte migration	2,43	3,37E-04	1,30E-02	CXCL6;CCL2;TGFB2;CXCL1;CXCL8;SLC7A6;PDE4D;ATP1B3;COL1A1;CCL7;ITGA2;APP;MSN;CD44;ICAM1;CX3CL1;CD34;MYH9;ITGB1
	GO:0050817	coagulation	2,63	4,20E-04	1,55E-02	PRKAR1B;PROCR;COL1A1;ILK;ITGA2;FLNA;CD9;CLIC1;SERPINB2;EHD1;CD34;MYH9;CD59;VCL;C1GALT1C1;ACTN1
	GO:1902903	regulation of supramolecular fiber organization	2,61	4,65E-04	1,63E-02	TACSTD2;CCN2;TGFB3;MAP2;CAPG;FLNA;APP;DLC1;TMSB4X;ICAM1;CX3CL1;TMSB10;JMY;ARHGAP18;NCK1;CKAP2
	GO:0002237	response to molecule of bacterial origin	2,60	4,80E-04	1,63E-02	CXCL6;CCL2;ANKRD1;CXCL1;CXCL8;VIM;IL18;TLR2;PDE4D;CARD16;RIPK2;ICAM1;GNG12;CX3CL1;ADAM9;CASP1
	GO:0071604	transforming growth factor beta production	7,45	5,10E-04	1,67E-02	TGFB2;LTBP1;ITGB6;CX3CL1;CD34
	GO:0001503	ossification	2,46	6,12E-04	1,93E-02	TNC;CCN1;TGFB2;RRAS2;VCAN;CCN2;ID3;TGFB3;TPM4;TUFT1;COL1A1;ILK;JAG1;CLIC1;COL6A1;EPHA2;BMP2
	GO:0031032	actomyosin structure organization	3,21	6,80E-04	2,06E-02	ANKRD1;TACSTD2;CCN2;TGFB3;ECT2;FRMD6;CNN2;DLC1;MYH9;TNFAIP1;ITGB1
	GO:0050878	regulation of body fluid levels	2,22	7,48E-04	2,19E-02	AKR1B1;PRKAR1B;PAM;FLI1;PROCR;COL1A1;PPAT;ILK;ITGA2;FLNA;CD9;CLIC1;SERPINB2;EHD1;CD34;MYH9;CD59;VCL;C1GALT1C1;ACTN1
	GO:0030048	actin filament-based movement	3,66	8,21E-04	2,27E-02	VIM;TPM4;DSC2;PDE4D;FRMD6;FLNA;MYL6;MYH9;ACTN4
Up-regulated genes in 3D	GO:1901615	organic hydroxy compound metabolic process	5,12	0	0	SLC27A2;DHCR24;CAT;SRD5A2;PECR;LDHD;FDX1;IDI1;GALK1;FDFT1;APOE;SLC7A11;HPN;RBP4;TTC39B;PTEN;ALDH3A2;ACAT2;PON1;MVK;HSD17B7;MOGAT3;DGAT2;FAH;LCAT;PAH;ADH1C;PLCD1;ABCA1;HMGCR;CYP7A1;DHRS3;SREBF1;TTPA;DDT;APOC1;ABAT;ACOX2;IP6K3; CEBPA;PSAT1;APOA2;SORD;AKR1C4;EPHX2;ADH6;PCK2;DAO;CYP4F3;CYP4A11;TTR;CYP4F2;FASN;ADH1A;DIO1;ABCB11;NR0B2;ALDH2; INSIG1;APOA1;HMGCS2;GPAM;CYP27A1;ADH4;MOGAT2;CYP39A1;AKR1B10;TKFC;SULT1E1
	GO:0044282	small molecule catabolic process	6,65	0	0	SLC27A2;PECR;AMDHD1;LDHD;CRYL1;ECHDC2;PON3;GALK1;RIDA;MLYCD;APOE;TST;EHHADH;GALE;HPD;THNSL2;GLUL;ADHFE1;PTEN;ACMSD;ALDH3A2;ACOT4;IRS1;HADH;ACAT2;PON1;GPT;NT5E;GLUD1;DPYS;CTH;FAH;FUT6;GLYCTK;PAH;ALDOB;ECI1; BDH1;ALDH6A1;AGXT2;IVD;PRODH2;ALDH1L1;LONP2;CYP7A1;AMT;FABP1;DCXR;HAAO;CYP4F11;ABAT;GCSH;ACOX2;GPT2;SORD;MAT1A;HAL;PCK2;DAO;SARDH;FTCD;CYP4F3;CYP4A11;PIPOX;CYP4F2;ALDH2;ADH4;CYP39A1;AKR1B10;TKFC;PKLR;AGXT;UPB1;SULT1E1;OTC;UROC1;TAT
	GO:0016053	organic acid biosynthetic process	5,37	0	0	SLC27A2;ACSL3;SEPHS2;HACD3;PECR;INSR;GALK1;MLYCD;NR1H3;THNSL2;GLUL;ACMSD;DDIT4;BHMT2;GPT;GAMT;GLUD1;HPGD;PHGDH;CTH;ALDH8A1;FBP1;PAH;ALDOB;CBR1;AGXT2;GATM;CYP7A1;FADS2;CPS1;APOC1;HAAO;ABAT;ACOX2;GPT2;PSAT1;GCKR;APOC3;ACSM2B;AKR1C4; EPHX2;ACSM5;CYP2C8;MLXIPL;FASN;FADS1;ABCB11;RGN;INSIG1;TRIB3;CYP27A1;CYP39A1;PKLR;CYP2C9;AGXT;UPB1;OTC; CYP1A2
	GO:0006631	fae y acid metabolic process	6,13	0	0	SLC27A2;ACSL3;HACD3;PECR;CRYL1;ECHDC2;PON3;MLYCD;EHHADH;NR1H3;THNSL2;ALDH3A2;ACOT4;IRS1;HADH;ACAT2;PON1;HPGD;PDP2;DGAT2;ECI1;CBR1;IVD;LONP2;CYP7A1;FADS2;SREBF1;FABP1;CYP4A22;APOC1;CYP4F11;CYP2D7;ACOX2;APOC3;ACSM2B;AKR1C4;EPHX2;ACSL1; PCK2;CYP4F3;CYP4A11;SLC27A3;ACSM5;ACSF2;CYP2C8;MLXIPL;CYP4F2;FASN;FADS1;RGN;INSIG1;TRIB3;GPAM;CYP2C9; GSTA1;CYP1A2;CYP2B6;CYP2A7;CYP2A6
	GO:0006732	coenzyme metabolic process	4,93	0	0	ACSL3;GSTO1;INSR;GALK1;MLYCD;SLC23A2;IDH1;ACMSD;ACOT4;DDIT4;MVK;BHMT2;SLC23A1;NT5E;GAMT;NNT;PDP2;DGAT2;FBP1; ALDOB;GPHN;HMGCR;ALDH1L1;DCXR;HAAO;CYP4F11;COQ10A;GLYAT;GCKR;PEMT;MAT1A;ACSM2B;VNN3;PANK1;ACSL1;FTCD;ACSM5; ACSF2;PIPOX;MLXIPL;CYP4F2;FASN;RGN;ASPDH;HMGCS2;GPAM;PKLR
	GO:0006520	cellular amino acid metabolic process	6,07	0	0	CARS2;BPHL;SEPHS2;AMDHD1;RIDA;SLC7A11;TST;HPD;THNSL2;GLUL;MSRA;ADHFE1;ACMSD;GCLM;BHMT2;HNF4A;GPT;GAMT;GLUD1; PHGDH;DPYS;CTH;FAH;PAH;ALDH6A1;AGXT2;IVD;PRODH2;GATM;AMT;MMUT;CPS1;HAAO;PEPD;ABAT;GCSH;GPT2;PSAT1;GLYAT;PEMT; MAT1A;HAL;DAO;SARDH;FTCD;NQO1;PIPOX;AGXT;UPB1;OTC;UROC1;TAT
	GO:0008202	steroid metabolic process	5,56	0	0	SLC27A2;DHCR24;CAT;SRD5A2;FDX1;IDI1;FDFT1;APOE;TTC39B;ACAT2;PON1;MVK;HSD17B7;DGAT2;LCAT;NR1I2;PRLR;ABCA1;HMGCR;CYP7A1;EGR1;SREBF1;APOC1;ACOX2;CEBPA;APOA2;RORC;AKR1C4;EPHX2;TFCP2L1;CYP2C8;FASN;ABCB11;NR0B2;INSIG1;APOA1;HMGCS2;GPAM; HSD11B1;CYP27A1;CYP39A1;CYP2C9;SULT1E1;CYP1A2;CYP2B6;CYP2A6
	GO:0042737	drug catabolic process	7,09	2,66E-15	2,83E-13	CAT;CTSH;RIDA;HPD;NT5E;NNT;DPYS;FAH;PAH;BDH1;NR1I2;AMT;GCSH;CYP2D7;PCK2;DAO;CYP2C8;PIPOX;ALDH2;CYP2C9;SULT1E1; CYP1A2;TAT;CYP2B6;CYP2A7;CYP2A6
	GO:0019216	regulation of lipid metabolic process	4,08	7,22E-15	6,82E-13	ACSL3;WDR81;IDI1;ABCB4;MLYCD;FDFT1;F2;APOE;NR1H3;IDH1;TTC39B;IRS1;MVK;HNF4A;PDP2;DGAT2;ABCA1;HMGCR;LONP2;CYP7A1; EGR1;SREBF1;FABP1;APOC1;GPLD1;APOA2;RORC;APOC3;EPHX2;ACSL1;CYP4A11;MLXIPL;FASN;FADS1;RGN;INSIG1;APOA1;TRIB3;HMGCS2;GPAM;HSD17B13;THRSP
	GO:0062012	regulation of small molecule metabolic process	4,21	2,58E-14	2,19E-12	KAT2A;IDI1;INSR;GCGR;MLYCD;FDFT1;APOE;SLC7A11;NR1H3;TTC39B;ACMSD;CLYBL;IRS1;DDIT4;MVK;PDP2;DGAT2;FBP1;HMGCR;LONP2; CYP7A1;EGR1;SREBF1;FABP1;PPP1R3B;APOC1;MST1;GPLD1;GCKR;RORC;APOC3;EPHX2;NQO1;MLXIPL;FASN;RGN;INSIG1;TRIB3;GPAM
	GO:0055088	lipid homeostasis	7,13	2,88E-14	2,22E-12	USF2;ABCB4;APOE;NR1H3;MTTP;TTC39B;HNF4A;DGAT2;LCAT;ABCG8;ABCA1;CYP7A1;ACOX2;CEBPA;APOA2;GCKR;APOC3;EPHX2;APOM; MLXIPL;INSIG1;APOA1;GPAM;CYP39A1
	GO:0009410	response to xenobiotic stimulus	4,61	3,90E-14	2,76E-12	BPHL;SRD5A2;NQO2;GSTO1;ABCB4;PON3;SERPINF1;DDIT4;CMBL;HNF4A;AKR7A3;HOMER2;PHGDH;DHODH;GSTA4;FMO3;NR1I2;CES3; EGR1;CPS1;ABAT;CYP2D7;GLYAT;RORC;ACSM2B;EPHX2;ACSL1;PCK2;CYP2C8;NQO1;CYP2C9;CYP1A2;CYP2B6;CYP2A7;CYP2A6
	GO:0042180	cellular ketone metabolic process	5,65	9,28E-14	6,07E-12	SRD5A2;MLYCD;SLC7A11;NR1H3;ACMSD;IRS1;PDP2;DGAT2;HMGCR;LONP2;CYP7A1;EGR1;SREBF1;FABP1;APOC1;CYP4F11;COQ10A;APOC3;AKR1C4;NQO1;MLXIPL;CYP4F2;RGN;INSIG1;TRIB3;ADH4;AKR1B10;CYP2B6
	GO:0016042	lipid catabolic process	4,20	2,81E-13	1,71E-11	SLC27A2;SRD5A2;PECR;ECHDC2;MLYCD;APOE;EHHADH;IDH1;ALDH3A2;IRS1;HADH;ACAT2;PLCD1;ECI1;IVD;LONP2;CES3;CYP7A1;FABP1; CPS1;APOC1;ACOX2;GPLD1;APOA2;APOC3;PCK2;CYP4F3;CYP4A11;CYP4F2;ENPP2;APOA1;GBA3;CYP39A1;AKR1B10;SULT1E1;CYP1A2
Up-regulated genes in 3D	GO:0006720	isoprenoid metabolic process	6,86	7,82E-13	4,43E-11	PECR;IDI1;FDFT1;APOE;RBP4;ALDH3A2;MVK;DGAT2;ALDH8A1;HMGCR;DHRS3;APOA2;APOC3;AKR1C4;APOM;TTR;APOA1;HMGCS2;ADH4;AKR1B10;CYP2C9;CYP1A2
	GO:0006638	neutral lipid metabolic process	6,80	9,34E-13	4,96E-11	CAT;APOE;NR1H3;MOGAT3;DGAT2;APOH;SREBF1;FABP1;CPS1;ANG;APOC1;GPLD1;APOA2;APOC3;ACSL1;PCK2;RGN;INSIG1;APOA1;GPAM;MOGAT2;THRSP
	GO:0006790	sulfur compound metabolic process	3,88	1,51E-12	7,53E-11	ACSL3;GSTO1;MLYCD;SLC7A11;TST;IDH1;MSRA;ACOT4;GCLM;MVK;BHMT2;GAMT;PHGDH;PDP2;CTH;DGAT2;GSTA4;HS3ST3B1;OPLAH; MMUT;CPS1;CHPF;GLYAT;PEMT;MAT1A;ACSM2B;ACSL1;ACSM5;ACSF2;PIPOX;FASN;HMGCS2;GPAM;AGXT;SULT1E1;GSTA2;GSTA1
	GO:0015711	organic anion transport	3,43	2,58E-12	1,22E-10	SLC27A2;ACSL3;SLC22A9;ABCB4;APOE;SLC7A11;SLC23A2;SLC25A15;MTTP;CA2;SLC17A4;MAP2K6;MFSD2A;SLC23A1;SLC2A2;SLCO1B1; SLCO2B1;STARD10;SLC25A13;ABCG8;ABCA1;FABP1;APOC1;ABAT;SLC38A4;APOA2;APOC3;AKR1C4;ACSL1;CYP4A11;SLC27A3;SLC25A18; CYP4F2;CA5A;AQP9;ABCB11;SLC22A7;APOA1;SLCO4C1;SLC38A3;AGXT;THRSP
	GO:1901568	fae y acid derivative metabolic process	5,71	4,47E-12	2,00E-10	ACSL3;PON3;PON1;HPGD;DGAT2;BDH1;CBR1;CYP4A22;CYP2D7;AKR1C4;EPHX2;ACSL1;CYP4F3;CYP4A11;CYP2C8;CYP4F2;FASN;FADS1; HMGCS2;CYP2C9;CYP1A2;CYP2B6;CYP2A7;CYP2A6
	GO:0010876	lipid localization	3,66	1,67E-11	7,10E-10	SLC27A2;ACSL3;SLC22A9;ABCB4;APOE;NR1H3;MTTP;RBP4;TTC39B;PON1;MAP2K6;MFSD2A;SLCO1B1;DGAT2;LCAT;SLCO2B1;STARD10; APOH;ABCG8;ABCA1;ABCA5;FABP1;TTPA;APOC1;APOA2;APOC3;AKR1C4;ACSL1;CYP4A11;SLC27A3;APOM;CYP4F2;AQP9;ABCB11;APOA1; THRSP
	GO:0006091	generation of precursor metabolites and energy	3,24	2,89E-11	1,17E-09	CAT;FDX1;PPP1R1A;NQO2;INSR;GCGR;GALK1;IDH1;IRS1;DDIT4;AKR7A3;NNT;PHGDH;COX6A2;FBP1;ADH1C;SLC25A13;ALDOB;BDH1; PPP1R3C;DHRS3;PPP1R3B;HAAO;GYS2;CEBPA;COQ10A;GCKR;GPX2;AKR1C4;ADH6;SLC25A18;NQO1;MLXIPL;ADH1A;ALDH2;XDH;RDH16; HMGCS2;ADH4;PKLR;CYP1A2
	GO:0043648	dicarboxylic acid metabolic process	6,59	6,12E-11	2,36E-09	SRD5A2;AMDHD1;SLC7A11;IDH1;GLUL;ADHFE1;ACMSD;ACOT4;GCLM;GLUD1;PRODH2;ALDH1L1;HAAO;GPT2;GRHPR;HAL;FTCD;UROC1; TAT
	GO:0005996	monosaccharide metabolic process	3,84	5,38E-10	1,99E-08	KAT2A;CRYL1;GSTO1;INSR;GCGR;GALK1;MLYCD;SLC23A2;GALE;RBP4;IRS1;SLC23A1;DGAT2;FUT6;FBP1;GLYCTK;SLC25A13;ALDOB;PPP1R3B;DCXR;MST1;GPLD1;GCKR;SORD;RORC;PCK2;RGN;TKFC;PKLR
	GO:0097006	regula- on of plasma lipoprotein particle levels	6,99	8,25E-10	2,92E-08	ACSL3;APOE;MTTP;PON1;DGAT2;LCAT;ABCA1;HMOX1;CES3;ABCA5;APOC1;GPLD1;APOA2;APOC3;APOM;APOA1
	GO:0015849	organic acid transport	3,56	1,74E-09	5,91E-08	SLC27A2;ACSL3;SLC22A9;ABCB4;APOE;SLC7A11;SLC23A2;SLC25A15;SLC17A4;MAP2K6;MFSD2A;SLC23A1;SLCO1B1;SLCO2B1;STARD10; SLC25A13;FABP1;ABAT;SLC38A4;AKR1C4;ACSL1;CYP4A11;SLC27A3;SLC25A18;CYP4F2;AQP9;ABCB11;SLC38A3;AGXT;THRSP
	GO:0046486	glycerolipid metabolic process	3,01	2,26E-09	7,38E-08	ACSL3;CAT;KLB;PIP4K2A;APOE;NR1H3;PTEN;IRS1;PON1;PIK3C2G;MFSD2A;MOGAT3;DGAT2;LCAT;STARD10;APOH;ETNPPL;SREBF1;FABP1; CPS1;ANG;APOC1;IP6K3;PCYT2;GPLD1;APOA2;PEMT;APOC3;ACSL1;PCK2;ENPP2;RGN;INSIG1;APOA1;GPAM;MOGAT2;THRSP
	GO:0043574	peroxisomal transport	7,53	3,41E-09	1,07E-07	SLC27A2;CAT;PECR;MLYCD;EHHADH;PEX16;IDH1;ACOT4;LONP2;ACOX2;EPHX2;DAO;PIPOX;AGXT
	GO:0071825	protein-lipid complex subunit organization	9,28	3,61E-09	1,09E-07	ACSL3;APOE;MTTP;PON1;LCAT;ABCA1;ABCA5;APOC1;APOA2;APOC3;APOM;APOA1
	GO:0051188	cofactor biosynthetic process	3,58	1,02E-08	2,98E-07	ACSL3;INSR;GALK1;MLYCD;SLC7A11;ACMSD;GCLM;DDIT4;PDP2;FBP1;ALDOB;OPLAH;GPHN;MMAB;HAAO;COQ10A;PSAT1;GCKR;MAT1A; PANK1;ACSL1;MLXIPL;FASN;RGN;ASPDH;PKLR;CYP1A2
	GO:0016999	antibiotic metabolic process	4,86	1,23E-08	3,48E-07	CAT;SRD5A2;AMDHD1;IDH1;ST6GAL1;NNT;ADH1C;AKR1C4;ADH6;HAL;PCK2;FTCD;ADH1A;ALDH2;ADH4;AKR1B10;SULT1E1;UROC1;CYP1A2

Figure S6. After 4 days of culture, transcriptomic analysis revealed a down-regulation of pro-inflammatory genes (red) and an up-regulation of xenobiotic metabolism enzymes (blue) in PHH cultured in 3D, as compared to 2D cultures. Differentially expressed genes had fold change >2 and p<0.05. Selected Go terms were the top 30 most enriched found in Webgestald online.

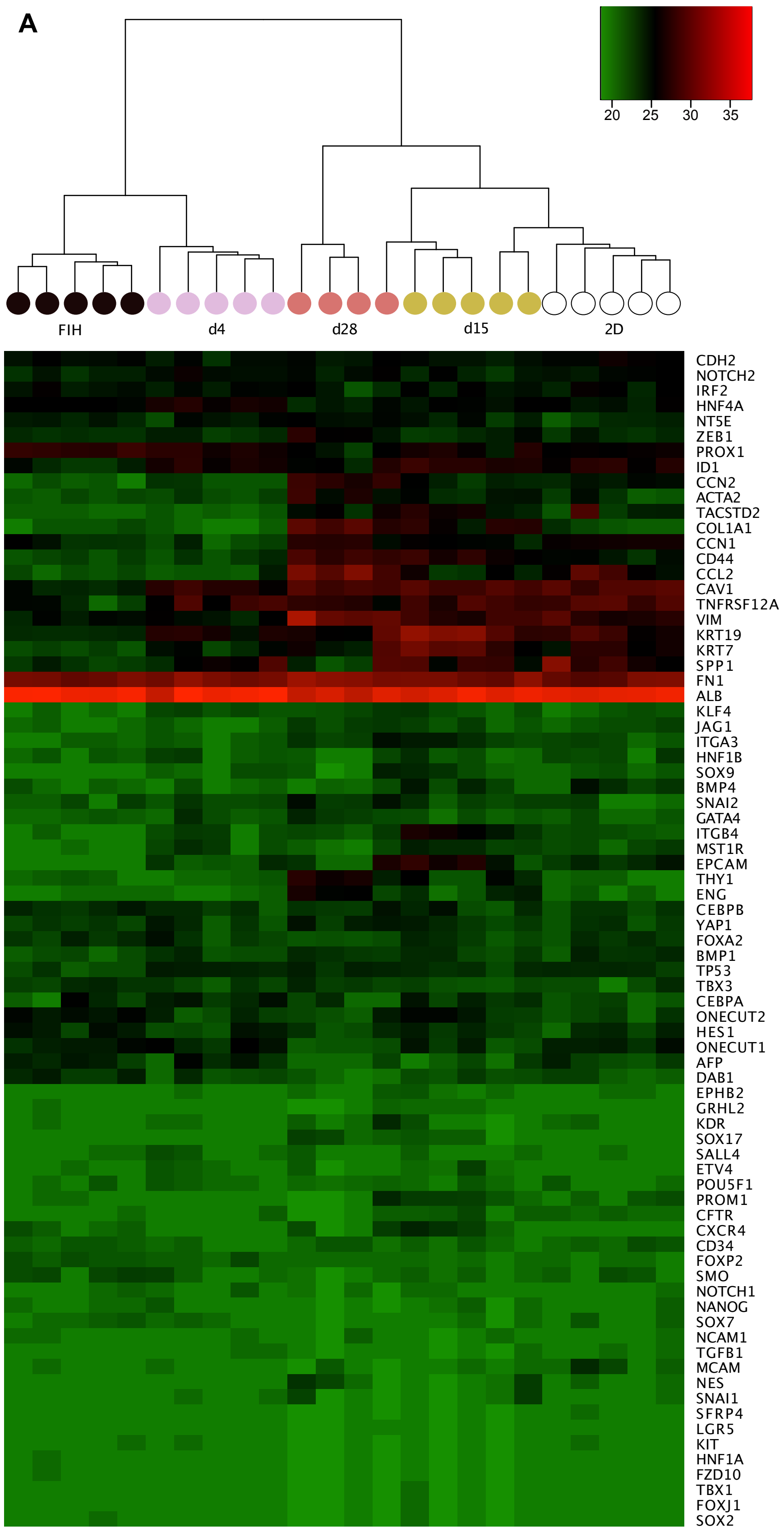

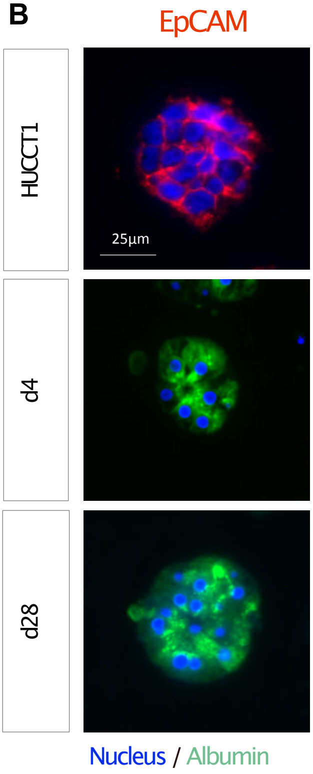

Figure S7. A. Heatmap of genes expressed by stem cells and mesenchymal cells in FIH (black), PHH cultured in 2D (white) and in 3D at day 4 (d4, pink), 15 (d15, yellow) and 28 (d28, red). *Albumin* expression (Alb): control of hepatic differentiation. **B.** Immunostaining of EpCAM (red), Albumin (green) and nuclei (blue) in the positive control HUCCT1 cells and in PHH spheroids at d4 and d28.

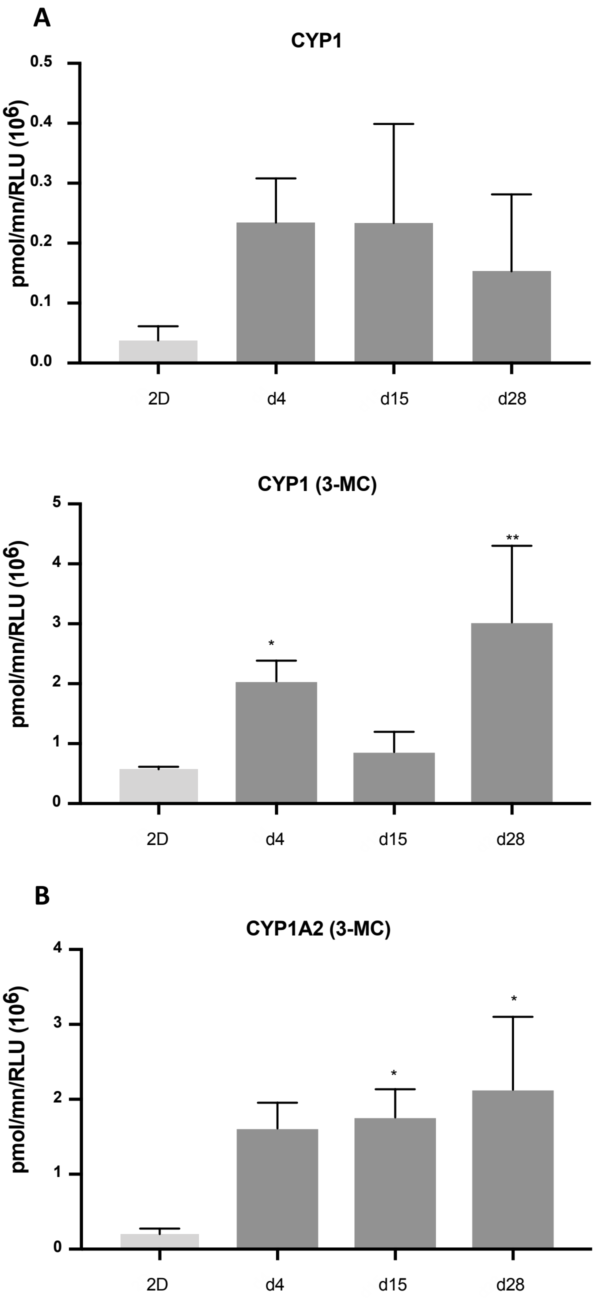

Figure S8. A. Basal CYP1 (EROD) activity or after 3-methylcholantrene induction (5 µM, 24h) in PHH cultured in 2D (light grey) or in 3D at d4, 15 and 28 (dark grey). **B.** CYP1A2 (MROD) activity after 3-methylcholantrene induction (5 µM, 24h) in PHH cultured in 2D (light grey) or in 3D at d4, 15 and 28 (dark grey). (Mean ± SD of triplicate, * $p<0.05$, ** $p<0.005$, Two-tailed Student's t-test, GraphPad Prism v.7.0).
